# CATER: Combined Animal Tracking & Environment Reconstruction

**DOI:** 10.1126/sciadv.adg2094

**Published:** 2023-04-21

**Authors:** Lars Haalck, Michael Mangan, Antoine Wystrach, Leo Clement, Barbara Webb, Benjamin Risse

**Affiliations:** ^1^ Institute for Geoinformatics and Institute for Computer Science, University of Münster, Heisenbergstraße 2, 48149 Münster, Germany.; ^2^ Department of Computer Science, University of Sheffield, Western Bank, Sheffield S102TN, UK.; ^3^ Research Center on Animal Cognition, Center for Integrative Biology, CNRS - Université Paul Sabatier - Bât 4R4, 169, avenue Marianne Grunberg-Manago, Toulouse 31062, France.; ^4^ School of Informatics, University of Edinburgh, Crichton St, Edinburgh EH8 9AB, UK.

## Abstract

Quantifying the behavior of small animals traversing long distances in complex environments is one of the most difficult tracking scenarios for computer vision. Tiny and low-contrast foreground objects have to be localized in cluttered and dynamic scenes as well as trajectories compensated for camera motion and drift in multiple lengthy recordings. We introduce CATER, a novel methodology combining an unsupervised probabilistic detection mechanism with a globally optimized environment reconstruction pipeline enabling precision behavioral quantification in natural environments. Implemented as an easy to use and highly parallelized tool, we show its application to recover fine-scale motion trajectories, registered to a high-resolution image mosaic reconstruction, of naturally foraging desert ants from unconstrained field recordings. By bridging the gap between laboratory and field experiments, we gain previously unknown insights into ant navigation with respect to motivational states, previous experience, and current environments and provide an appearance-agnostic method applicable to study the behavior of a wide range of terrestrial species under realistic conditions.

## Teaser

A tool is developed to obtain high precision quantification of animal behavior filmed in the wild with a freely moving camera.

## INTRODUCTION

For more than 50 years (*1*), researchers have sought technologies to accurately quantify the behavior of animals in their natural habitats. Advances in imaging technology, computer vision, and machine learning have enabled a variety of breakthroughs in the computational analysis of animal behavior in recent years (*2*–*5*) with different tracking systems developed for model organisms such as *Drosophila melanogaster* flies (*6*, *7*), larvae (*8*, *9*), *Caenorhabditis elegans* (*10*), zebrafish (*11*), and mice (*12*, *13*).

Animal tracking approaches are usually divided into two categories, namely, pose estimation and positional detections over time (*14*). In particular, deep learning algorithms have enabled fully automatic pose estimations from video recordings. Prominent examples are DeepPoseKit (*15*), DeepLabCut (*16*), and LEAP (*17*). Recently, extensions of these algorithms have improved the applicability of these algorithms for multi-animal pose estimation (*18*, *19*). For positional detection of animals over time, both conventional computer vision and deep learning algorithms have been used. For example, Ctrax (*20*), idTracker (*11*), Multi-Worm Tracker (*10*), and FIMTrack (*9*) are well-known examples for conventional tracking algorithms using background subtraction and dedicated foreground identification strategies. In contrast, machine learning–based detection algorithms usually use (fully) convolutional neural networks to identify the objects of interest such as the idTracker.ai (*21*), Mouse Tracking (*22*), fish CNNTracker (*23*), and anTraX (*24*).

Because the difficulty of visual tracking typically increases with the complexity and variability of the scenery (*25*), these systems have primarily been developed for controlled laboratory conditions (*18*, *19*, *21*, *26*). The difficulty is further aggravated for small animals like insects, which do not provide visually distinctive features (limiting feature-based methods such as deep learning approaches), while navigating unpredictably in a cluttered and highly ambiguous environment (preventing background modeling and requiring camera motion compensation) (*27*). Moreover, the lack of unique features and visual ambiguities challenge the capabilities of state-of-the-art tracking algorithms, which often use discriminative correlation filters, Siamese correlation, or transformer-based machine learning architectures (*28*). To provide a general purpose in-field animal tracker, three fundamental challenges must be addressed. First, animals must be detected consistently over time, even when occupying few pixels, providing low contrast, and across periods of occlusion. Second, these detections must be linked and warped into a camera motion compensated trajectory in a common frame of reference. Third, trajectories must be embedded in an environment reconstruction, allowing researchers to relate trajectories to environmental features, such as obstacles. At the time of writing, there is no software that addresses all three challenges in an end-to-end pipeline, facilitating long-range and unconstrained in-field animal tracking (table S1) (*25*).

The visual tracking of individually foraging ants provides an excellent example of these challenges. These insects are comparatively small and often visually indistinguishable from each other and their complex natural habitat (background). On the other hand, ants exhibit highly complex navigation strategies such as path integration (PI) and visual memory mechanisms, which are best studied under natural conditions in the field (*29*). Desert ant behavior is currently quantified over longer ranges, either using hand annotation with reference to a preinstalled grid (*30*, *31*) or by following individuals with differential Global Positioning System (GPS) device (*32*). Complementary analysis of the fine-scale movement patterns in small areas [from fixed cameras stationed at the nest, e.g., (*33*, *34*) or using data from trackballs placed at discrete locations, e.g., (*35*)] have inspired new hypotheses regarding how visual memories are learned (*36*) and used (*37*), respectively. However, many questions remain unresolved. For example, how quickly can visual route memories be learned? How is foraging behavior affected by motivational state and environmental interactions? What are the underlying control strategies that govern motion during navigation? To tackle these questions, there is a need for in-field quantification techniques that enable unconstrained measurement of microscale behavior in the animal’s natural habitat.

In this work, we bridge the gap between laboratory experiments and in-field studies by demonstrating how new visual tracking methods provide unique insights into the natural navigation behavior of ants. Our core contribution is a combined solution for unconstrained visual animal tracking and environment reconstruction framework [called Combined Animal Tracking & Environment Reconstruction (CATER)], capable of detecting small targets in video data captured in complex settings while embedding their locomotion path within a high-resolution environment reconstruction in moving camera conditions. This has been achieved by:

1) An unsupervised probabilistic animal detection mechanism, which identifies the object of interest in the images based on motion alone, making its appearance agnostic, robust to occlusions and camera motion, and capable of localizing even tiny insects in cluttered environments.

2) A dense and globally optimized environment reconstruction algorithm, which can process millions of frames to calculate a high-resolution image mosaic from multiple freely moving camera videos.

3) A unified and highly parallelized tracking framework combining the detection and reconstruction algorithm to generate full frame rate animal locations projected onto the very high-resolution environment reconstruction.

4) Integrated routines and graphical interfaces for user interactions to enable efficient corrections and manual annotations to contextualize the behavioral measurements.

5) The application of this framework to obtain a uniquely high-resolution (time and position) dataset describing the entire foraging history of individual desert ants in their complex desert-shrub habitat.

Evaluations of our framework yield excellent spatial and temporal accuracy. We demonstrate its applicability in a setting beyond the capabilities of existing approaches by successfully integrating 1.8 million images from multiple recordings into a unique high-resolution environment reconstruction while achieving a median tracking accuracy of 0.6 cm evaluated on more than 300,000 manually annotated images. CATER enables us to obtain detailed trajectories, which revealed a number of original insights into ant navigation.

## RESULTS

### Data collection

**Fig. 1.**
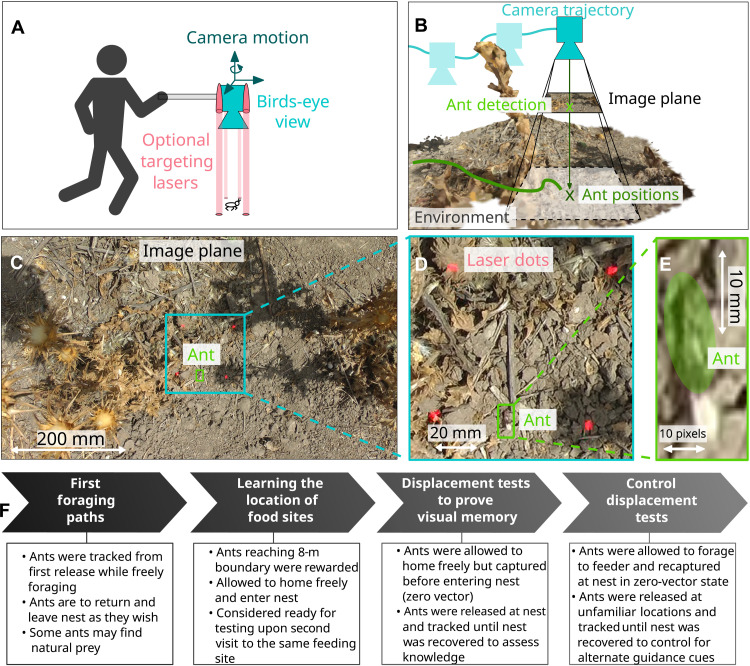
Data capture and visual in-field tracking overview. (**A**) Ant paths were recorded using an off-the-shelf camera augmented with downward facing laser pointers to aid camera/ant alignment. (**B**) From these recordings, camera trajectories and ant detections are estimated and combined to compute the ant position (animal trajectory) with respect to the environment reconstruction. (**C** to **E**) Example image from the dataset at full resolution and zoomed in around the ant. (**F**) Flow chart outlining the experimental protocol to assess the minimal learning and memory requirements for visual route following in desert ants (“Data capture” section in Supplementary Text provides additional information).

Our test scenario is a study of the foraging ontogeny of desert ants (*Cataglyphis velox*). For video recording, an off-the-shelf camcorder (Panasonic HDC-TM900) was used to capture uncompressed 1920 by 1080 video recordings at 50 frames per second. A custom-made camera rig with a 1.5-m horizontal arm was used to capture video from directly above the ant (approximately 1 m) without the experimenter disturbing the forager (Fig. 1A). To simplify the capture process, four standard red laser pointers were secured around the camera pointing downwards to create a visible light pattern on the ground to aid camera/ant alignment (Fig. 1, A and B). Given that ants blend in with the background and only cover approximately 30 by 6 pixels in the image (Fig. 1C), they are barely visible for human observers and can only be identified in zoomed croppings (Fig. 1, D and E). This clearly presents a major challenge for any existing tracking algorithm.

We followed each individual ant (having marked them) from the first time that they exited the nest (Fig. 1F). The ant was free to forage in any direction, interact with other ants and vegetation, and return to the nest as it wished. Only when its foraging path reached approximately 8 m from the nest was it provided with a food morsel. Foraging ants will more or less rapidly establish visually guided idiosyncratic routes if they discover a site with a regular food supply (*30*, *31*). Following their second homeward trip from the same feeding site, individuals were subjected to a series of displacement tests to assess their visual memory performance given minimal experience (details are in “Data capture” section in Supplementary Text). The resultant dataset totals 151 videos (1.8 million individual frames) documenting the complete foraging history and displacement trials of 14 foragers in their natural habitat (details of complete dataset are in “The ant ontogeny dataset” section in Supplementary Text).

### Tracking and reconstruction framework

**Fig. 2.**
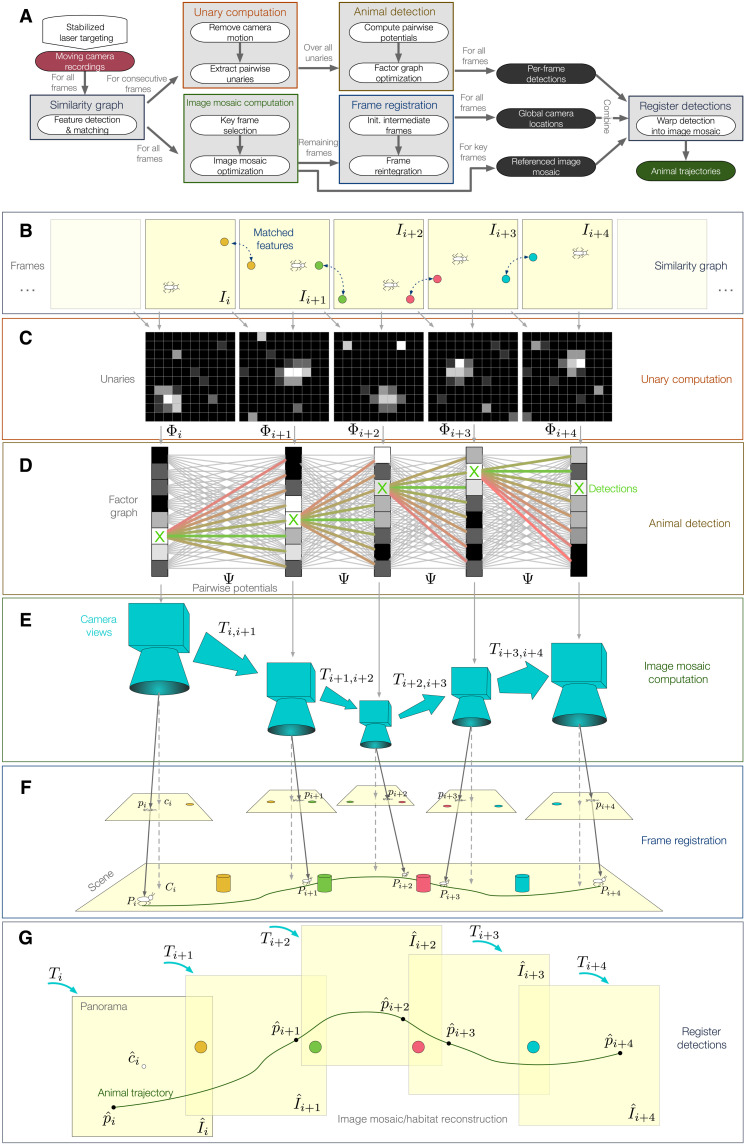
Insect tracking and environment reconstruction framework. (**A**) Overview of the detection and tracking framework. Consecutive frames *I_i_* are used (**B**) to compute camera motion compensated unaries Φ*_i_* (**C**), which are connected by pairwise potentials Ψ to build a factor graph (**D**). In-frame ant detections *p_i_* are then computed using the factor graph and projected based on the camera views (**E**) into a unified reference system ${\hat{p}}_{i}$ using globally referenced frame transformations *T_i_* (**F**) to project the images ${\hat{I}}_{i}$ into a unified image mosaic (**G**) to generate animal tracks.

Our framework consists of several modules processed in an interleaved fashion, as summarized in Fig. 2A. For all frames *I_i_*, a similarity graph is generated by extracting and matching features across all frames from all videos (Fig. 2B). The frames and resultant transformations are then passed to two parallelized tracking and reconstruction tasks.

In the first task, matches from consecutive images are used to calculate pairwise transformations *T*_*i*,*i*+1_ (*38*). These transformations are used to compensate the camera motion and to extract motion residuals between consecutive images called unaries Φ_i_ (Fig. 2C; details are in “Tracking and reconstruction algorithm” section in Materials and Methods). That is, each unary represents the probability distribution of animal movement as a two-dimensional (2D) heatmap. Assuming a moving ant in the majority of the frames, the motion residuals can be interpreted as 2D probability distributions of potential ant locations. These locations **p_i_** = (*x*, *y*) are connected by a motion model (i.e., pairwise potentials Ψ_*p_i_* → *p*_*i*+1__ between two consecutive detections **p_i_** and **p_i+1_**) to encourage smooth pixel transitions and temporal consistency for all possible locations in each frame. By combining all unaries and pairwise potentials into a factor graph (which is a graphical representation of the underlying belief network; Fig. 2D), the most probable ant locations $p_{i}^{*}$ for all frames *i* = 1, …, *T* can be computed by using the globally optimal max-sum algorithm (*39*)

$$\arg\max_{p_{i}^{*},\cdots ,p_{T}^{*}}E(p_{1},\cdots ,p_{T}|I_{1},\cdots ,I_{T})=\sum_{i=1}^{T}{\text{Φ}}_{i}+\sum_{i=1}^{T-1}{\text{Ψ}}_{p_{i}\to p_{i+1}}$$

This global probabilistic inference formulation can be solved independently of the object and background appearance, does not require any initialization or manually labeled training data (unsupervised), and is capable of resolving ambiguities such as occlusions automatically.

In the second task, 2D environment reconstructions are extracted by identifying key frames from any number of video sequences and warping these frames *I_i_* into a unified image mosaic coordinate system ${\hat{I}}_{i}$ based on their pairwise transformations *T*_*i*,*i*+1_ (Fig. 2E). Subsequently, introduced drift is mitigated by minimizing an optimization problem using the transformations between all frames. Afterwards, all intermediate frames need to be reintegrated to enable full frame rate trajectories. This is done using geodesic interpolation followed by a refinement step resulting in dense transformations *T_i_* for all frames (Fig. 2F) (*40*). In the following, we refer to the resultant globally optimized image mosaic as environment reconstruction or map to emphasize that this mosaic recovers visual characteristics in two dimensions.

Last, ant locations **p_i_** need to be projected onto the image mosaic via *T_i_* to embed the trajectories into the environment reconstructions (Fig. 2G). The approaches are thus combined into a unified unsupervised tracking framework that yields high-accuracy paths and environmental maps without initialization or calibration routines.

In contrast to nonvisual tracking methods [such as telemetry (*41*)], our resulting dataset preserves all visual environment information, allowing post hoc augmentations that both improve the data quality (e.g., through human-in-the-loop tracker corrections) and allow emergent research questions to be tackled after data collection. In particular, labels can be added to individual frames (e.g., to note behavioral changes such as acquisition of food) or specific locations tagged in the background image mosaics (e.g., feeding sites). The entire framework as well as the interaction functionality is embedded into a unified graphical user interface and offers a variety of convenient and usable features and visualizations for fast data interaction (details are in “Tracking and reconstruction algorithm” section in Materials and Methods and fig. S2). 

### Tracking and reconstruction performance evaluation

Our object detection and tracking algorithm is appearance agnostic and can be used to detect all kinds of animals and artificial objects in a variety of different environments and lighting conditions (*39*). Here, we use this localization pipeline to successfully recover the position of ants in all 1.8 million images of our ontogeny dataset.

**Fig. 3.**
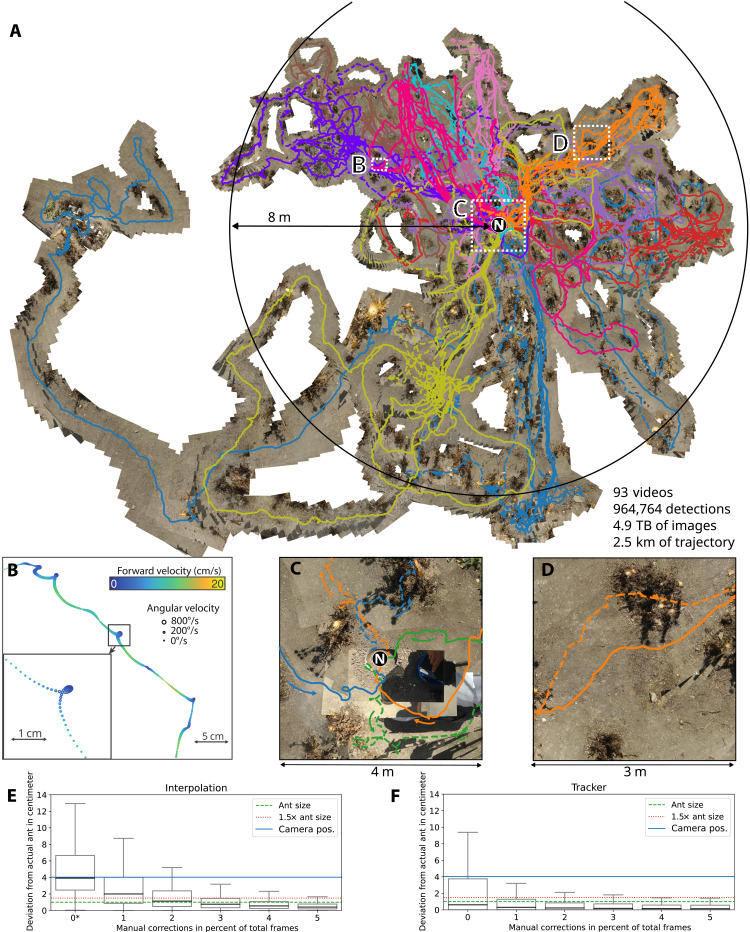
Reconstructing the foraging history of desert ants in the wild. (**A**) A unified high-resolution (41,248 by 30,351 pixels) top-down image mosaic incorporating 93 videos on which the recovered paths of their respective ants are plotted. (**B**) The 50-Hz temporal resolution allows accurate forward and angular velocities to be measured and fine-scale behavioral motifs such as loops to be mapped. (**C**) Behavior with respect to key locations [here, the first departures (dashed lines) and returns (solid lines) of three ants from the nest (N)] can be compared across individuals. (**D**) Environmental interaction with terrain features (here, bushes) can be recovered. (**E**) Deviation of tracking results per frame using linear interpolation between equidistant points. 0∗ stands for two points being the minimum for successful linear interpolation. (**F**) Deviation of tracking results per frame using the tracker. The points are selected on the basis of high deviation from the ground truth.

Our reconstruction pipeline registers background scenes to create high-resolution top-down image mosaics of the ant habitat on which the complete foraging paths of multiple ants can be plotted (Fig. 3A and fig. S6). Previous evaluation has shown that our registration mechanism can achieve millimeter spatial accuracy and a median angular error of 3° (*40*). The resultant data are particularly unique: The high-temporal resolution (50 Hz) allows observation of fine-scale behavior across the animals complete foraging path in their natural habitat (Fig. 3B); the embedding of paths into a shared frame of reference allows high-resolution comparison of behaviors within and across individuals (Fig. 3C). Moreover, the ability to add labels while reviewing the videos permits retrospective analysis; i.e., conditions of interest such as interactions with vegetation or other ants (Fig. 3D) do not need to be explicitly recorded by the experimenter at the time of data collection, provided that they can be observed in the video data.

Figure 3 (E and F) compares the performance of the tracking algorithm with respect to the amount of manual positional estimates versus a linear interpolation between the same amounts of evenly spaced points. We evaluated the tracking performance using 307,956 manually annotated images and achieved a median accuracy of 0.6 cm. Integrating just 1% of these manual annotations into the globally optimized probabilistic inference task further improves the accuracy to 0.31 cm median accuracy. In contrast, the results using linear interpolation needs more points to achieve similar median accuracy. We note that this is the accuracy of the true ant position rather than an estimate of position of the tracking device (e.g., GPS unit) as a proxy for the animal position and that the behavioral statistics do not change substantially if manually specified corrections are included to the probabilistic inference task (cf. fg. S5). Performance is particularly robust for homing ants that travel quickly over open terrain while carrying a clearly visible food morsel. These results are in line with previous evaluations of the detection mechanisms in which a localization accuracy above 96% was achieved on the publicly available Small Targets within Natural Scenes dataset (*39*). On the basis of accurate (in-frame) localizations, the accuracy of camera motion compensated trajectories can be estimated based on the precision of the image mosaic algorithm, which has been estimated under laboratory conditions in a separate publication (*40*).

### Tracking yields insights from detailed route comparisons

A crucial and novel outcome of our processing framework is that it allows successive routes of the same ant to be precisely registered through anchoring on the terrain. This allows us to probe how accurately an ant can follow a route using visual memory alone after minimal experience. This was tested by tracking individual ants until they had returned twice from the same feeding site. The first-time ants were permitted to enter the nest and deposit the food; the second time, they were captured just before nest entry and (still holding the food item) released back at the feeding site. This eliminates PI information, and as these ants do not use chemical trails, it allows us to test visual navigation in isolation.

**Fig. 4.**
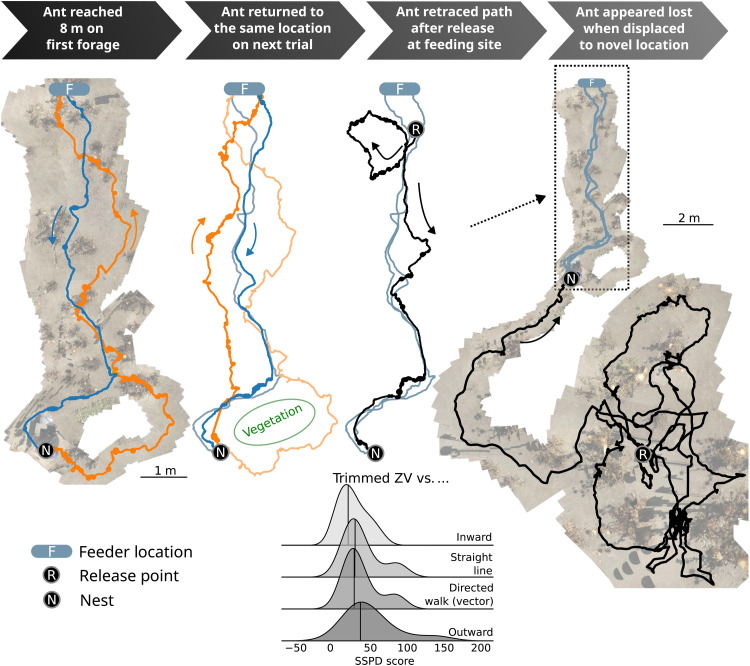
Tracking results indicate that homeward route memories are acquired rapidly. The foraging history of an individual ant is shown (left to right). On the second return (second from left), it is captured before nest entry and displaced back to the feeding site and is able to retrace the same homeward route without path integration (PI) information (third from left). Ridgeline plot (below): The similarity to its previous route is higher than to a straight line [as measured by symmetrized segment-path distance (SSPD) as defined in the section "Trajectory Preprocessing" in Materials and Methods"], a directed walk with realistic noise, or to its outward path. Control (far right): Displacement to a location not previously experienced results in lengthy search indicating the absence of any long-range cues to the nest position.

An example of the entire foraging history of an individual ant as it progressed through our experimental protocol is provided in Fig. 4. This example clearly demonstrates that after just two visits to the same feeding site, the displaced ant was capable (after an initial search) of retracing its homeward path, a result replicated by all 14 ants tested in this way. There is notable overlap in each return path. Excluding any initial search segment (i.e., trimmed), we quantitatively compared the homing route taken after displacement to the same ant’s immediately previous homeward route, for all ants (see “Trajectory processing” section in Materials and Methods). We observed a close similarity in the precise path taken; substantially more similar than would be expected if the ant was just trying to run in the nest direction (e.g., if attracted by a beacon or using a local vector), or if it was retracing its outward route (Fig. 4, ridgeline plot). The same ants were subsequently displaced to a location, which they had not previously experienced (3 to 4 m from the nest in a perpendicular or 180° direction from their feeding location), and were observed to engage in search, only able to home if their search path crossed over a previous inward route (Fig. 4, right).

These data provide strong evidence that *C. velox* desert ants need to experience a route no more than twice (and only once successfully reaching the nest) to have committed it to memory with sufficient reliability to be able to use it to return home in the absence of PI information. This supports previous reports of one-time route learning either in isolated instances (*31*) or over short route segments (*42*, *43*).

We note that the ant shown in Fig. 4 foraged to the 8 m boundary and returned with food on its first outing, as did two other ants; that is, they did not appear to perform “learning walks.” This lack of recent experience of the nest surrounds (all ants that had foraged in the previous 2 days were excluded) did not affect their homing ability either on their first homeward trip (Fig. 3C and “Homing performance of ants without exploratory paths” section in Supplementary Text) or in their route displacement test for which they ranked second, third, and fifth of the 14. Nine of the ants made at least one initial exploratory path (returning without food) in the nest vicinity (explorations: mean = 2.2, SD = 1.9; see “The ant ontogeny dataset” section in Supplementary Text). However, unlike the usual characterization of learning walks (*33*, *36*, *44*–*46*), we saw no consistent growth in the duration (mean/SD, first: 27/23 s and last: 36/53 s) or distance covered (mean/SD, first: 0.60/0.32 m and last: 0.64/0.34 m) in these paths. Although the angular coverage increased over trips (mean/SD, first: 98°/37° and last: 191°/78°), foraging was generally restricted to a specific angular sector (Fig. 4 and “Characterization of initial exploratory forages” section in Supplementary Text). This puts into doubt an assumption frequently made in recent computational models of ant visual memory (including our own) (*47*–*51*) that all foraging ants use learning walks to acquire views from multiple directions and a range of distances around the nest as a basis for reliable homing behavior.

### Reconstructions enable habitat interaction studies

The ability to reexamine the behavior of ants in the context of the merged video information allows us to identify the effect of habitat features. Upon locating food, ants will return directly to the nest (using PI) and are then assumed to return to the location where the food was found to scavenge further (using a vector memory, that is, inverting the PI information) (*52*). Our data reveal clear differences between paths taken when returning to either a known feeding location or the nest implying different strategies depending on the motivational context. Outward paths of ants to the 8 m boundary gradually increased in their similarity, directness, and speed of travel over successive journeys indicating that they are refined over time, which contrasts with homeward paths that were fixed, direct, and travelled quickly from their first instance (“Differences in outward and inward route learning” section in Supplementary Text).

**Fig. 5.**
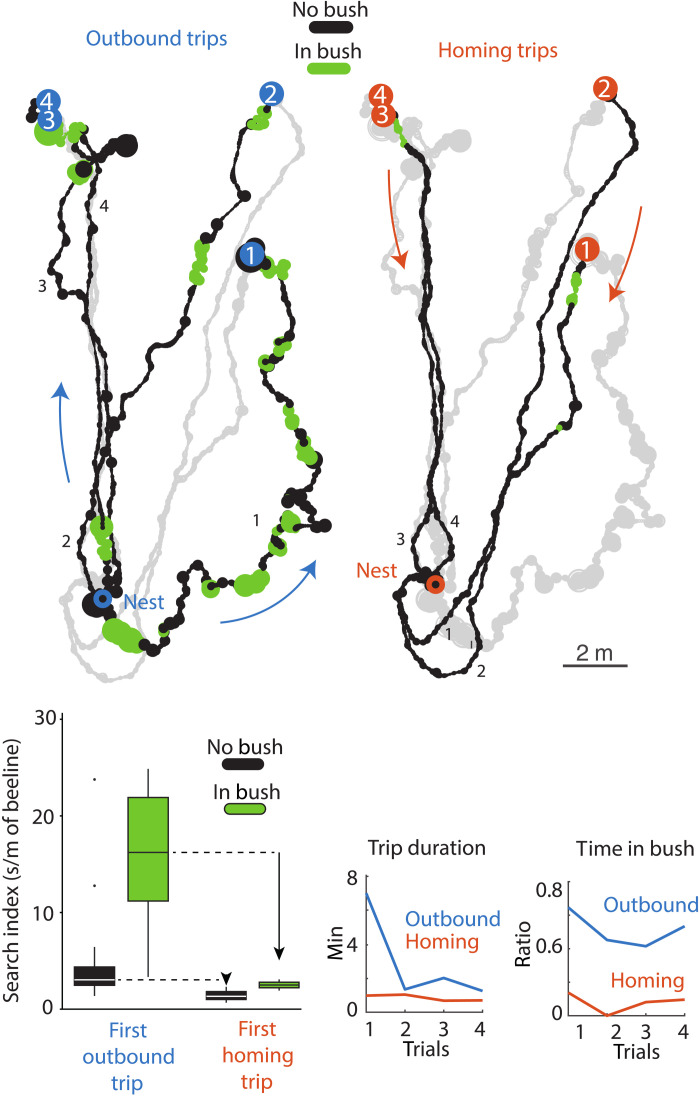
Environment reconstruction enables post hoc habitat interaction studies. The first four foraging trips of an ant to the 8 m boundary are shown with sections spent within vegetation highlighted in green. The ant entered bushes significantly more during its outward (13 bushes, 334 s) than its homing trips (two bushes, 10 s). The boxplot depicts the search index (a measure of amount of local searching; see “Trajectory processing” section in Materials and Methods for search index calculation) of the ant for route sections within bushes compared to those in open terrain for both the first outbound and first homing trip. Only on outward trips did the ant engage in search behaviors, and search increased within bushes. The duration and ratio of time spent in bushes shows a clear tendency to enter bushes on outbound trips versus a tendency to avoid bushes when homing (bottom right).

Closer examination shows that some ants entered bushes much more often on their outward trips than homing trips (ratio of time in bush; Fig. 5), suggesting different strategies driven by motivational state. Foraging ants may target bushes to locate prey (e.g., snail carcasses) or shelter from the sun, while homing ants aim to get home as quickly as possible and thus avoided the bushes (Fig. 5). This is supported by the fact that, when in a bush, foraging (outbound) ants slow down considerably and spent time searching (higher search index), whereas homing ants (which rarely entered a bush) continued moving at their usual rapid pace through bushes (Fig. 5, boxplot). Incorporation of such adaptive strategies into computational models will be important in maintaining their explanatory power in more realistic settings.

### High spatiotemporal data yields mechanistic insights

**Fig. 6.**
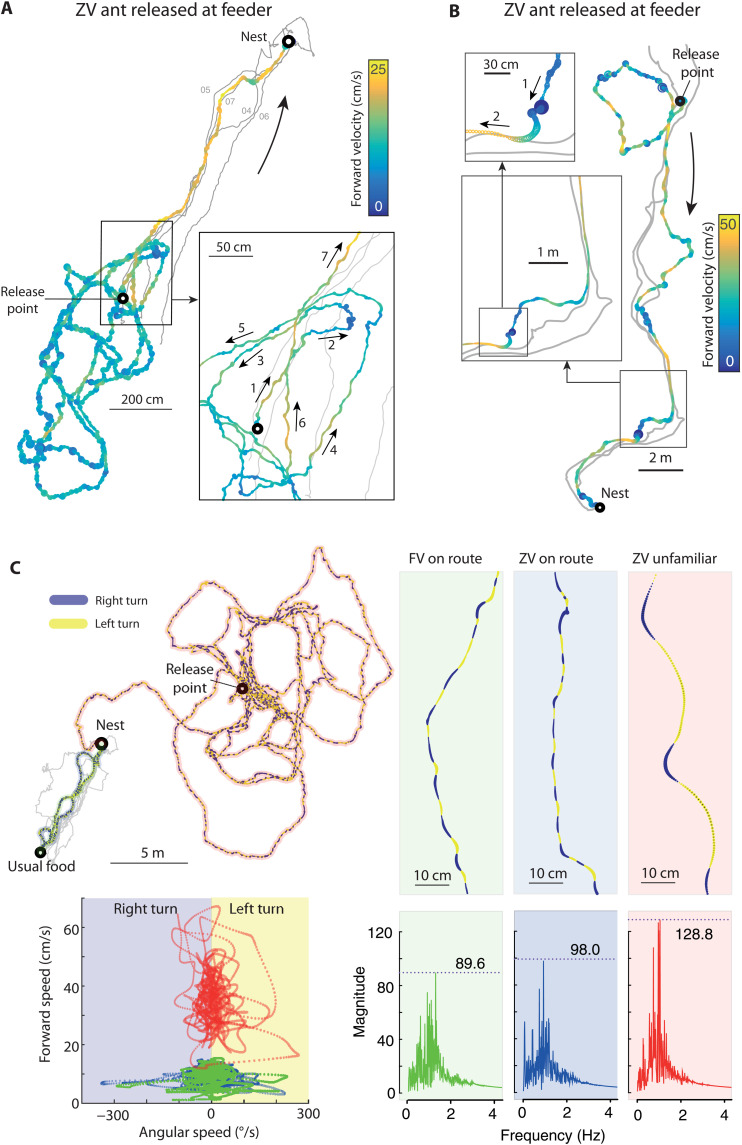
Mechanistic insight from high spatiotemporal data resolution. (**A** and **B**) Tracks of ants after displacement to the feeding site, with forward velocity indicated by color and angular velocity by line thickness. Previous tracks are in gray. In (A), the ant’s initial search is slow with extensive rotation compared to the straight and fast movement once the familiar route is found. Inset, zoomed section of search: A similar transition to faster, straighter motion can be seen in detail each time the ant faces in the direction of the route (arrows 1, 4, 6, and 7), suggesting moments of visual recognition. (B) Zooming in on another ant mid-route, we can identify a “scan,” where forward velocity falls to zero and angular velocity exceeds 500°/s (arrow 1) immediately preceding its regaining of the route (arrow 2). (**C**) Comparison of movement dynamics for an ant homing from her usual feeding location with [full-vector (FV) on route, green] or without [zero-vector (ZV) on route, blue] a PI vector or when released in an unfamiliar area without a PI vector (ZV unfamiliar, red). For this ant, there is a clear increase in forward velocity in the unfamiliar environment (bottom left). Highlighting right (blue) and left (yellow) turning reveals regular lateral oscillations. Zooming in (top right) shows that oscillation amplitude increases markedly in the unfamiliar environment. The Fourier spectrum of the angular velocity (bottom left) shows similar frequency of oscillations (around 1 Hz) but variation in their regularity (magnitude) across conditions.

Our dataset provides the spatiotemporal resolution (50Hz positioning at centimeter accuracy) necessary for revealing the mechanisms that underpin guidance behavior in insects. For example, a host of competing hypotheses have been proposed regarding the encoding, storage location, and use of visual route memories in insects (*51*, *53*, *54*). One way to tease apart these models is to reveal the precise circumstances under which navigating insects recognize their location (e.g., the change in distance and orientation at which a view is recognized is likely to be specific to an individual model). Our data demonstrates local bursts of forward speed when the ant trajectory becomes aligned in a “familiar” direction (Fig. 6, A and B), that is, along a route it has previously taken toward the nest. We can thus infer the locations and conditions under which ants recognize a previously traversed route. We also observe instances of scanning movements (sharply reduced forward speed and increased angular velocity) (*55*) immediately following moments when the ant strays from a previously traversed route (Fig. 6, A and B), allowing us to infer when there is a failure of recognition. Unexpectedly—and not captured by recent models (*37*, *51*, *54*)—some ants moved much faster and displayed less local meander when searching in a completely unfamiliar environment (Fig. 6B, bottom left panel). This suggests that slowing and scanning behaviors may be triggered by sharp (phasic) changes in familiarity, rather than the tonic familiarity level of the current location, as has previously been assumed.

We also observe, in ants moving freely in natural conditions, the expression of regular lateral oscillations (Fig. 6C). These have previously been seen in laboratory conditions (*56*) and in tethered ants running on a trackball in the field (*35*, *57*) but have not been reported for ants moving over natural terrain, as they are hard to perceive by eye. The oscillation frequency is remarkably conserved (around 0.9 Hz) across conditions, but their regularity (magnitude of the Fourier peak) is modulated by the familiarity of the visual environment (Fig. 6C and fig. S10). Oscillations are most regular with zero-vector (ZV) ants in unfamiliar environment (Fig. 6C and fig. S10). This fits nicely the idea that an intrinsic neural oscillator is constantly at play in navigating ants but that both PI [available in full-vector (FV) ants] and visual scene recognition (available when on the familiar route) modulate these oscillations and thus interfere with their regularity (*35*, *57*) by adding externally driven left and right motor commands (*37*, *56*, *58*). Overall, this shows how detailed kinematic data at the natural scale can shed light on the mechanisms at play during an ecological task.

## DISCUSSION

The ability to track animal behavior over the natural range of foraging distances has been transformative in our understanding of navigation behavior. For bees, it provided definitive evidence that dance communication provides new foragers with a vector to find the food location (*59*). In bats, it revealed the pin-point accuracy with which animals could target a particular foraging location (a single tree) over large distances (kilometers) (*60*). Even in laboratory rats, the use of a larger arena was a critical factor in the discovery of grid cells (*61*). However, until now, substantially increasing the range while maintaining the spatial and temporal resolution of laboratory tracking conditions (*18*) was not possible, particularly under natural conditions where the computer vision challenges are substantially increased (*25*, *27*).

Here, we have shown that, using a single camera to follow a foraging animal, we can reconstruct both detailed tracks and natural habitats. CATER is a general visual tracking and reconstruction framework capable of extracting dense movement patterns of unmarked animals irrespective of their size (even down to a few pixels), camera motion (both static and moving), or setting (controlled laboratory to natural habitats). It combines algorithms for small object detection (*39*) and full frame rate–optimized reconstruction (*40*) into a novel and fully automatic tracking framework, which is applicable to extremely challenging datasets. As a result, animal locations and habitat reconstructions from potentially multiple videos are incorporated in a global reference frame, enabling a wide range of behavioral quantifications and studies. We show its application to data from freely foraging ants in a complex desert-shrub habitat, consisting of multiple videos from handheld cameras with more than 1.8 million frames and a total of more than 2 km of insect tracks. The tracking module achieved centimeter-level precision without any manual correction, with tracks embedded in a shared reconstructed image mosaic. Note that this processing pipeline is designed to provide high-precision trajectories of individual animals followed by the camera. It has therefore been developed for tracking a single animal of interest with no built-in multi-animal tracking capabilities and can only be used in a post hoc fashion (no real-time tracking). The resulting output documents the entire foraging history of desert ants, as they learned to navigate their local environment at unprecedented spatiotemporal resolution.

As a result of these methodological advances, we have already gained important new insights into the visual navigation capabilities of ants. The combination of accuracy within tracks and consistent registration between tracks has revealed that ants follow previous routes very tightly. From instantaneous speed data, we could directly confirm that slowing and scanning—which have been assumed to indicate uncertainty about the route—are associated directly with points at which the ant has deviated from its previous route, and clear increases in speed can be observed when the route is regained. We found that visual memory for the inbound path is obtained very rapidly and appears equally accurate along the whole course of the path, and accuracy in return is independent of time spent in “learning walks” around the nest. We believe this is indicative of “one-shot” learning of visual scenes by the ant. By contrast, the outward path evolves more gradually, under the influence of alternative foraging instincts such as approach to bushes. The ability to retrospectively annotate the video footage to identify such environmental influences and relate them to behavior is an important contribution of our approach. Last, the high temporal resolution provided by video tracking provides additional mechanistic insight by revealing a steady underlying oscillation in the ant’s paths, observed under all conditions (foraging, homing, and searching), which appears to be an intrinsic active strategy controlling visual steering.

Robust motion tracking as a behavioral readout is fundamental to fields from neuroscience (*62*) through genetics (*63*, *64*) to medicine (*65*) and ecology (*5*, *25*). Although this study focused on tracking insects from handheld camera footage, the methods described are widely generalizable. For example, drones are increasingly being used to capture video of different animals in distinct settings [e.g., large terrestrial mammals (*66*, *67*) or fishes in shallow water (*68*)], and the data produced are remarkably similar to that presented here and thus can be processed in our framework to obtain detailed paths projected onto their territory. Similarly, multicamera systems and structure from motion pipelines could be added to capture the 3D structure of animal habitats (*69*), which combined with new biologically realistic sensing (*70*) and brain models (*51*, *54*, *71*) would allow hypotheses to be verified in real-world conditions. In the future, we will integrate additional features such as multi-animal tracking and real-time user feedback during recording. The applications of such general tracking methods thus extend beyond behavioral analyses and include habitat management (*72*) and conservation (*2*, *66*).

## MATERIALS AND METHODS

### Tracking and reconstruction algorithm

#### Overview

The proposed method consists of several interacting modules that are explained in more detail below. Transformations between consecutive images are used to rectify the camera motion in the unary computation stage or between all frames in the image mosaic computation stage to find key frames and to generate a 2D background representation. Following the unary computation, animal detections are computed using factor graph optimization. These animal coordinates in per-frame pixel space are transformed using the global camera locations resulting from camera registration and following the image mosaic generation. An abstract overview of the complete framework is outlined in Fig. 2A.

##### Unary computation and detection

Consecutive images are transformed using their pairwise transformation. To minimize the reprojection error and thus the noise in the resulting unaries, we use homographies in this stage, since they have the highest degree of freedom. Given images *I_i_*, *I*_*i*+1_ and their estimated homography *H*_*i*,*i*+1_, image *I*_*i*+1_ can be warped into the same coordinate system as *I_i_* using

$${\hat{I}}_{i+1}=H_{i,i+1}^{-1}\;\cdot I_{i+1}$$ (1)

effectively removing camera motion between those two images. The remaining motion is calculated by subtracting the warped image Î and *I*

$$D_{i}=|{\hat{I}}_{i+1}-I_{i}|$$ (2)

The difference *D_i_* contains only moving objects like the moving animal as well as plants moving in the wind, shadows, and noise due to errors in the estimation of the transformation or due to parallax.

The objective of the following optimization problem is to find a probable and temporally consistent path of a single animal through the full video. The optimization problem is formulated as a probabilistic inference problem estimating animal positions **p_i_** = (*x_i_*, *y_i_*) for all frames *i* ∈ {1, …, *T*} for a total of *T* images given the frame differences as observations D = {*D*_1_, …, *D_T_*}. The energy function to be maximised is defined as follows (*39*)

$$E\;(p|D)=\left[\sum_{i=1}^{T}\text{Φ}\;(p_{i}|D_{i})\right]+\left[\sum_{i=1}^{T}\text{Ψ}\;(p_{i},p_{i+1})\right]$$ (3)

The first functional Φ(·) defines the unary potential and is described in terms of the remaining motion *D_i_* weighted by a centered Gaussian distribution to amplify regions in the center and reduce motion cues at the edge of the images

$$\text{Φ}(p_{i}|D_{i})=N(\mu ,\sigma^{2})\;\cdot D_{i}$$ (4)

for some predefined mean μ and variance σ^2^ [see (*39*) for details].

The second functional Ψ(·) defines the pairwise potential and encourages temporal consistency by coupling detections between consecutive images

$$\text{Ψ}(p_{i},p_{i+1})=N(p_{i+1}|p_{i},\sigma^{2})$$ (5)

Intuitively, the optimization maximizes areas of motion while enforcing smooth transitions by limiting the step size between images. The functional from Eq. 3 problem is maximized using factor graphs (*39*).

##### Image mosaic computation and registration

To decrease the computational complexity of the image mosaic computation and reduce visual artifacts, the first step is a key frame selection. Starting with the first image as the first key frame, the following key frames are selected using multiple metrics. A candidate must have enough overlap with the previously selected key frame and should have a small reprojection error (i.e., the error of the transformation) with a high amount of good quality feature points covering a substantial portion of the image. Exhaustive feature matching is done on all key frames to find recurring places in the full scene. For consecutive key frames, transformations are estimated warping them into them same coordinate system.

For this stage of the pipeline, similarities are used instead of homographies, encoding scale, rotation around the viewing direction, and a translation for a camera that is directed perpendicular toward the ground. The goal of the image mosaic generation is to find the best globally consistent reconstruction of the environment, whereas the goal of the unary computation was to minimize noise between consecutive images. Because of the additional degrees of freedom, the later optimization of the transformation is more unstable and often results in unusable image mosaics.

Let F = {*I*_1_, …, *I_N_*} be the set of all *N* images and let

$$F=\{F_{i}\subset R^{2}|i\in \{1,\ldots ,N\}\}$$

be their corresponding feature vectors, where *F_i_* is a set of 2D feature points.

Given *K* key frames $\{{\tilde{I}}_{1},\ldots ,{\tilde{I}}_{K}\}\subset I$ and their corresponding pairwise similarity ${\tilde{T}}_{i,i+1}$ for a consecutive pair of key frames ${\tilde{I}}_{i}$ and ${\tilde{I}}_{i+1}$, we can define transformations with respect to the first key frame by a multiplication of the transformation matrices

$$\begin{array}{rl} {\tilde{T}}_{j} & =\prod_{i=1}^{j-1}{\tilde{T}}_{i,i+1}^{-1}\;\mathrm{for}\;2\leq j\leq N\\ {\tilde{T}}_{1} & =1 \end{array}$$ (6)

We call these matrices global transformations, as they project a certain frame into a common reference coordinate system. The multiplication leads to an error manifesting itself in a drift where points of revisiting do not align between earlier and later frames in the videos.

Subsequently, an optimization problem is solved, where common feature points in all frames are used to mitigate errors in the initial estimates

$$\begin{array}{c} \min J({\tilde{T}}_{1},\ldots ,{\tilde{T}}_{K})=\sum_{1\leq i\leq K}\sum_{i\leq j\leq K}\sum_{(f_{k},f_{l})\in {\tilde{M}}_{i,j}}\\ ∥({\tilde{T}}_{j}^{-1}{\tilde{T}}_{i})\;\cdot \;f_{k}-f_{l}{∥}_{2}^{2}+∥({\tilde{T}}_{i}^{-1}{\tilde{T}}_{j})\;\cdot \;f_{l}-f_{k}{∥}_{2}^{2} \end{array}$$ (7)

For feature matches ${\tilde{M}}_{i,j}\subset {\tilde{F}}_{i}\times {\tilde{F}}_{j}$ between key frames ${\tilde{I}}_{i}$and ${\tilde{I}}_{j}$, minimizing the symmetric reprojection error of the global transformations.

Given two key frames ${\tilde{I}}_{i}$ to ${\tilde{I}}_{j}$, this energy function reduces the difference between directly transforming from ${\tilde{I}}_{i}$ to ${\tilde{I}}_{j}$ by using ${\tilde{T}}_{i,j}$ and using ${\tilde{T}}_{i}$ to transform ${\tilde{I}}_{i}$ to the reference coordinate system and then using ${\tilde{T}}_{j}^{-1}$ to transform to frame ${\tilde{I}}_{j}$. The direction of this transformation can also be reversed leading to the above functional. These transformations can then be used to align the images to generate a common image mosaic.

With these optimized transformations of the key frames alone, animal detections in frames between two key frames cannot be projected onto the generated image mosaic. To calculate transformations for all frames, the omitted frames from the preceding key frame selection are reintegrated using geodesic interpolation and are subsequently refined using feature matches between the previous and next key frame, resulting in dense and accurate transformations *T_i_* for each frame *i* ∈ {1, …, *T*}. The optimization and subsequent reintegration and refinement can also be generalized to multiple videos by combining key frames of all videos in the optimization in Eq. 7. Reintegration and refinement is subsequently done for all videos simultaneously. Details for the optimization, reintegration, and multiple video extension can be found in (*40*). The registration process is illustrated in fig. S1.

##### Trajectory generation

The last step is to overlay per-frame detections onto the image mosaic, which can be achieved by matrix vector multiplication with the transformation estimate of the corresponding frame. Given per-frame detections **p_i_** = (*x_i_*, *y_i_*) for a frame *I_i_* for all *i* ∈ {1, …, *T*} and their respective transformations *T_i_* mapping them into the common coordinate system, we transform *p_i_* according to

$${\hat{p}}_{i}=T_{i}\;\cdot p_{i}$$ (8)

To generate the full animal trajectory, detections of all frames—intermediate and key frames—have to be used. This is possible, since we have one transformation for each frame and not only for the sparse set of key frames after performing reintegration and refinement. The described transformation can also be used to overlay camera trajectories onto the generated image mosaic by transforming points **c_i_** = (*w*/2, *h*/2) for images with a width of *w* and a height of *h* for all for all *i* ∈ {1, …, *T*}.

##### GUI, human interactions, and parallelization

The tracking and reconstruction algorithms are combined into a single framework that can be accessed via a graphical user interface (GUI). This GUI can be used to load and process the video frames and provides a variety of visualization strategies and user feedbacks to simplify the tracking process. The graphical interface can also be used to attach labels and provides a summary indicating the quality of the frames with respect to the tracking task. See fig. S2 for more details.

The method described in “Unary computation and detection” section supports two types of human interaction. First, the user can manually correct the animal position by manually clicking on correct animal positions. Note that because of the factor graph optimization, a single click can correct multiple frames (*39*). Second, the user can also easily add labels to a video to allow more complex behavioral analysis. For example, in this study, we added labels indicating the visibility of the ant, whether the ant was within shadows or bushes, plus the ant’s current state (foraging versus homing).

The computational time used to solve the optimization problem in Eq. 3 across all frames made manual corrections cumbersome. To alleviate this, after initial global optimization, videos were chunked into smaller subsections, which were processed in parallel to let the user see the impact of their manual correction more quickly. After revising the whole video, the full optimization can be performed again.

##### Trajectory preprocessing

The generated raw trajectory is smoothed by using a Savitzky-Golay smoothing filter (*73*) with a polynomial order of 3 and a window size of 50, which equals to 1 s in our recordings. Trajectories in pixel space on the generated image mosaics were scaled to real-world space in centimeters using an object with known scale.

Velocity metrics from the paths were obtained by first calculating “instantaneous velocities vectors” as the vectors connecting each two subsequent trajectory points. Forward velocity values were then obtained as the norm of the instantaneous velocity vectors (Euclidean distances between successive points). Angular velocity values were obtained as the angle between two subsequent instantaneous velocity vectors. Determining the sign of angular velocity (whether the ant is turning clockwise or counterclockwise) is intrinsically ambiguous. However, here, the frame rate (50 Hz) can resolve angular velocities up to 180°/0.02 s (i.e., 9000°/s), which is by far higher than the maximum angular velocity displayed by ants. Therefore, the sign of angular velocity values between two consecutive frames could be easily disambiguated by choosing the comparison (*n* → *n* + 1 or *n* + 1 → *n*) that yielded an angle smaller than 180°. Velocity values presented in Fig. 6 were smoothed using first a median filter (sliding window of 10 frames, 0.2 s, to remove aberration data) and then an average filter (sliding window of 10 frames, 0.2 s, to reduce noise). Absolute angular velocity values in Fig. 6 were converted linearly into dot thickness of a scatter plot.

Straightness of a trajectory is defined by the ratio of its total path length (sum of the length of all segments between consecutive trajectory points) divided by the distance between its start and end point as discussed in (*74*). Distance between trajectories is quantified using the symmetrized segment-path distance (SSPD), which is based on point to segment distances as described in (*75*) rather than dynamic time warping (DTW), which is based on point-to-point correspondences. SSPD is therefore less dependent on the velocity of the two compared trajectories. Higher velocity results in fewer points on the trajectory, which leads to higher distances when using DTW.

The search index presented in Fig. 5 corresponds to the time spent within a bush (or between bushes) divided by the Euclidian distance between the path’s entrance point in a bush and subsequent exit point (or the exit point of one bush to the entrance point of the next bush for between-bushes indexes). Directed random walks are defined by a realistic step size determined by the average velocity of the model species and an angle sample from a random distribution centered around the target angle determined by the angle from the current position to the nest. Directed walks are then generated with a same straightness as the corresponding trimmed zero vector path using the metric from above. This is done by repeatedly sampling random walks until a desired straightness is achieved.

## Supplementary Materials

### This PDF file includes:

Supplementary Text Figs. S1 to S11 Table S1 Legend for movie S1 References
