# Supplementary material for: CATER: Combined Animal Tracking & Environment Reconstruction
Source: Sci Adv. 2023 Apr 21;9(16):eadg2094. doi: 10.1126/sciadv.adg2094 (PMC10121171; doi:10.1126/sciadv.adg2094)
Supplement: Supplementary file 1 — Supplementary Text Figs. S1 to S11 Table S1 Legend for movie S1 References [file sciadv.adg2094_sm.pdf]

Science Advances
AAAS

Supplementary Materials for

CATER: Combined Animal Tracking & Environment Reconstruction

Lars Haalck *et al.*

Corresponding author: Benjamin Risse, b.risse@uni-muenster.de

Sci. Adv. **9**, eadg2094 (2023)
DOI: 10.1126/sciadv.adg2094

The PDF file includes:

Supplementary Text
Figs. S1 to S11
Table S1
Legend for movie S1
References

Other Supplementary Material for this manuscript includes the following:

Movie S1

Supplementary Text

Dataset

CATER has been evaluated using a dataset beyond the capabilities of existing tracking algorithms (called Ontogeny Dataset). Here we provide detailed information on how we captured the data and additional behavioral results.

<u>Data Capture</u>

Field Site & Experimental Species. The field study was conducted in an abandoned field on the outskirts of Seville, Spain (37°20’ N, 5°59’ W) between 5th and 31st July 2012. This region is home to the thermophilic ant species Cataglyphis velox whose foragers navigate using visual, rather than pheromone, cues (31). The site comprised a large flat area populated by low level foliage common to shrub-land including grassy tussocks and dried thistle bushes. A nest was located in an area where the vegetation formed a maze-like environment for foragers to navigate, and obscured distal visual cues such as trees or buildings. A physical barrier (plastic ring with internal height ~20cm) was installed around the nest to prevent foragers from exiting the nest without help from an experimenter. The foraging area surrounding the nest (circle of ~8m radius) was swept ahead of data capture to remove natural prey and to provide a clean surface for ants to forage (Figure S3).

Experimental Protocol. Ahead of data capture, experienced foragers were removed from the population by permitting their exit, but blocking return to the nest for two days. Subsequently, naive foragers that showed a strong urge to forage (e.g., attempting to climb the walls) were marked with enamel paint to allow re-identification over repeated journeys. When selected for tracking, marked ants were then carefully lifted over the barrier and gently placed on the ground by an experimenter. Returning ants were able to reach the nest unaided via a one-way earth ramp.

Painted and motivated foragers were tracked as they freely explored their nest surrounding in search of food. Foragers were free to return to the nest at any time. The first experimental intervention occurred when foragers reached the predefined perimeter approximately 8m from the nest where they were provided with a reward (either a cookie crumb or piece of meal-worm). Note that as ants were allowed to forage without restriction, feeding locations were unique to each ant. All ants readily carried their bounty back to the nest. The subsequent outward and inward paths of ants were recorded, with food rewards provided each time ants reached 8m boundary. The second experimental intervention occurred after ants had returned home from the same feeding location for a second time. These ants were captured just before entering the nest at which point their PI home vector will have expired (thus termed zero vector (ZV) ants). To test whether a single complete traversal of the inward route is sufficient for visual route recovery, ZV ants were transferred back to the feeding site in an opaque cylinder where they were released and tracked (ZVF trial). Two subsequent ZV displacement tests were conducted to ensure that route recovery was not a function of any other navigation cues. Specifically, ants were displaced to a ’semi-familiar’ location, approximately 90 degrees and 3-4m away from their normal homing

path (ZVSF trial), followed by a completely unfamiliar location approximately 180 degrees and 3-4m from their normal homing path (ZVOP trial). Note that between these trials ants had to complete an additional out and return path to the feeder to create the zero-vector condition.

Recording Hardware. For video recording an off-the-shelf camcorder was used to capture 1080p video recordings with 50 frames per second without compression (Panasonic HDC TM-900). A custom-made camera rig mounted to the camera on a 1.5m horizontal arm allowing video to be recorded from directly above the ant at a constant level height without the experimenter disturbing the foragers. To simplify the capturing process, we attached standard red laser pointers around the camera pointing to the ground providing. Thus, the experimenter simply had to keep the ant within the laser dot pattern which corresponds to the center of the image frame.

The Ant Ontogeny Dataset

We recorded the paths taken by 14 ants from their first foraging trip until they completed each of the displacement trials Figure S4 details the video data captured for every ant as they progressed from early unrewarded foraging paths (light green background), to foraging paths that extended to our 8m boundary where they were rewarded (bolder green background), before being subject to a series of displacement trials (blue background). We note that in some cases ants encountered natural prey within the 8m search space (peach background) and may have searched at more than one 8m feeding site ahead of testing (see notes column).

Notable points include: 14 ants were tracked until they had completed the ZVF trial, 13/14 also completed the subsequent ZVSF control, and 11/14 also completed the final ZVOP control; 7/14 ants returned to their first 8m feeding site on the next outward path, a further 5 returned to a 2nd site on successive outwards paths, and 2 moved onto a 3rd location; 3/14 ants found natural prey during their normal foraging; excluding these rewarded trials ants completed on average 2.2 (median=2, standard deviation=1.9) foraging paths in the local nest area before returning to the nest without reward.

For video analysis all videos were converted into image stacks using ffmpeg v4.2.4 (*https://ffmpeg.org/*) on Ubuntu 20.04 using the command:

ffmpeg -i video file.mp4 imgs/frame %08d.png

The tracker was run on all 151 videos. Manual positional corrections were added to 52 videos, including all 31 local search paths that did not lead the ants to natural prey or the 8m boundary, plus 8/14 ZVF trials. This ensured that the tracking position was within 1 body-length of the animal in every frame. We note that the majority of the remaining uncorrected videos are displacement trials in which they ant generally moves faster, in the open, and is carrying a cookie which improves automated tracking substantially. Indeed, analysis of the paths of the tracker for correct vs uncorrected ZVF trials shows no significant difference (Figure S5.)

In additional, contextual labels were added to many videos. Firstly, a label was added to all frames across all 151 videos indicating whether ants were carrying a food item (cookie or natural prey) or not, which we take as a proxy for motivation (foraging vs homing). For 70/151 videos (26/26 exploratory walks, 13/14 ZVF trials, and the foraging routes of 7 ants) labels were added

to indicate all frames in which ants entered a bush, entered a shadow or were invisible to the camera (e.g., obscured by a bush). Finally, for all 26 videos that could be considered exploratory walks, occurrences of scans, voltes, or pirouettes (*33, 45*) were added.

Behavioral Analysis & Results

CATER outputs both, image mosaic reconstructions of the environment and embedded trajectories of the animals. Several example routes and reconstructions can be found in Figure S6 and Section Supplementary Video provides a video demonstrating the combined tracking and reconstruction results.

Homing Performance of Ants Without Exploratory Paths

Figure S7A plots the final approach (the paths within 2m from the nest) of 3 ants that reached the 8m boundary on their first foraging trip in their subsequent return journey (colored paths). The final approaches of the first return from the 8m boundary of the 9 ants that completed an average of 2.2 exploration walks before reaching the 8m boundary (black paths) and thus should have more experience of the nest area are also shown. Note that paths have been rotationally aligned to ease visual comparison of directness Figure S7B plots the straightness and duration of the final approach for these groups. The ants that did not complete any runs before reaching the 8m boundary show no clear degradation in homing performance.

Characterization of Initial Exploratory Forages

Figure S8 plots the search time (top row), maximum distance (middle row), and accumulated angular coverage (bottom row) for each of the exploratory paths (those that did not reach 8m) for all ants tracked. Each ant is assigned a unique color and data-points of individuals are linked to allow easily comparison and observation of trends respectively. Single data points are added to the lower plot for ants that either reached the 8m boundary on the first forage, or found natural prey to assess whether they had explored around the nest during that first long forage. The time and distance of the observed explorations paths show no clear increase over successive trips and while there is a positive trend in the accumulated angular coverage, ants typically reached the 8m boundary without exploring an area beyond 200°.

Differences in Outward & Inward Route Learning

Figure S9 presents data showing the difference between outward and inward route learning in ants. In all panels, each ant is assigned a unique color and data-points of individuals linked to allow easily comparison and observation of trends respectively. Figure S9 (top panel) shows the accuracy with which individuals returned to the previous feeding site. Figure S9 (2nd row) shows the similarity of successive outward (left column - dashed lines) and successive inward routes (right column - solid lines) of ants as measured using the (SSPD Score - see Section Trajectory Processing). Figure S9 (3rd row) shows the straightness of successive outward (left column - dashed lines) and successive inward routes (right column - solid lines) of ants when compared to the direct line between their feeding site and the nest. That is a perfectly straight

path would score 1. Figure S9 (bottom row) shows the average speed of ants during successive outward (left column - dashed lines) and successive inward routes (right column - solid lines).

Comparison to State-of-the-Art

To demonstrate the novelty of our algorithm compared to several state-of-the-art detection algorithms as shown in Table S1. As illustrated, none of the existing algorithms is capable of addressing the three major challenges of in-field insect tracking. Moreover, we compared our system with existing image stitching object detection algorithms as shown in Fig. S11. As illustrated in Fig. S11 A, conventional image stitching algorithms fail to extract consistent image mosaics and Fig. S11 B demonstrates that background subtraction-based foreground segmentation strategies (here kNN as used in (76)) also fail to provide acceptable animal localizations. Fig. S11 C shows detection results of CATER compared to YOLOv5 (77) and Super-DiMP (78). Given the very small size of the insects, deviations >10 pixel can already lead to erroneous behavioral characteristics (see also Fig. 1 E).

Supplementary Figures

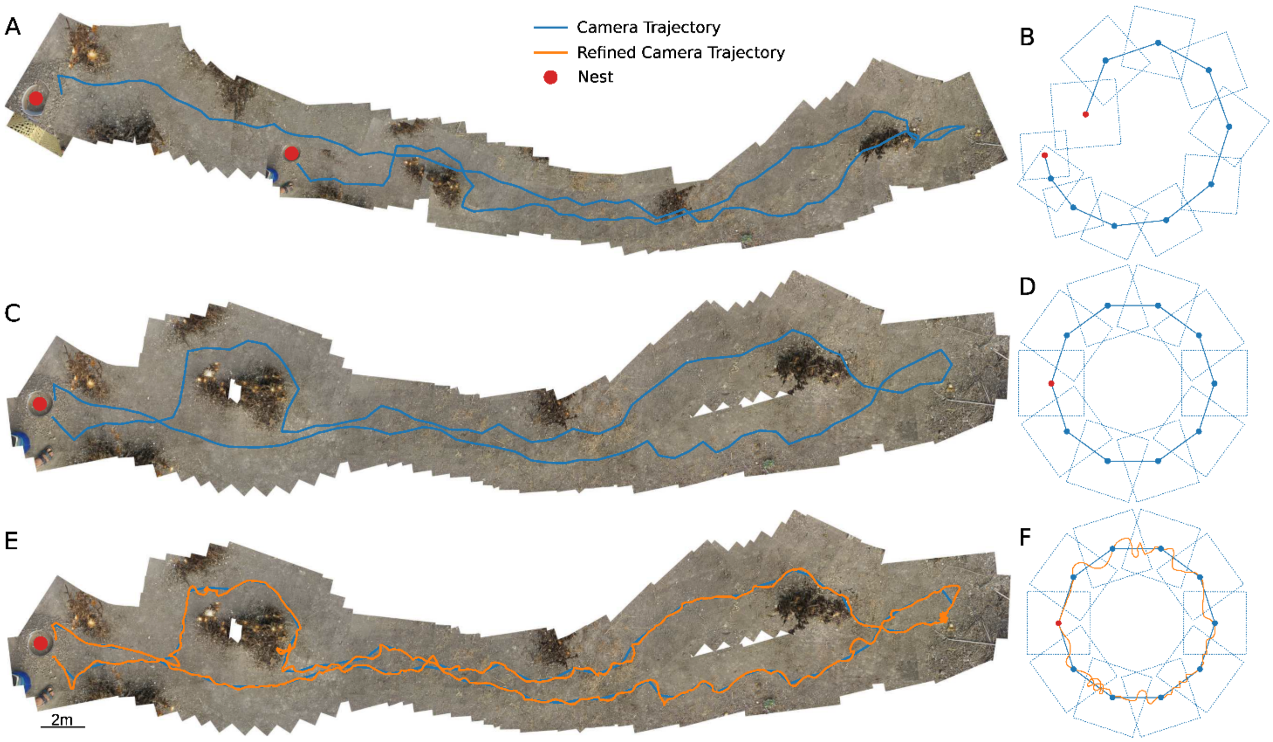

Fig. S1. Image mosaic generation and map refinement overview. Comparison of an image mosaic before **(A, B)** and after **(C, D)** optimization and after final refinement **(E, F)**. (A, B) before optimization the same nest at the start and end of the video do not align. (C, D) the nest aligns, closing the gap between start and end frame. (E, F) Refinement recovers tiny motion in between key-frames.

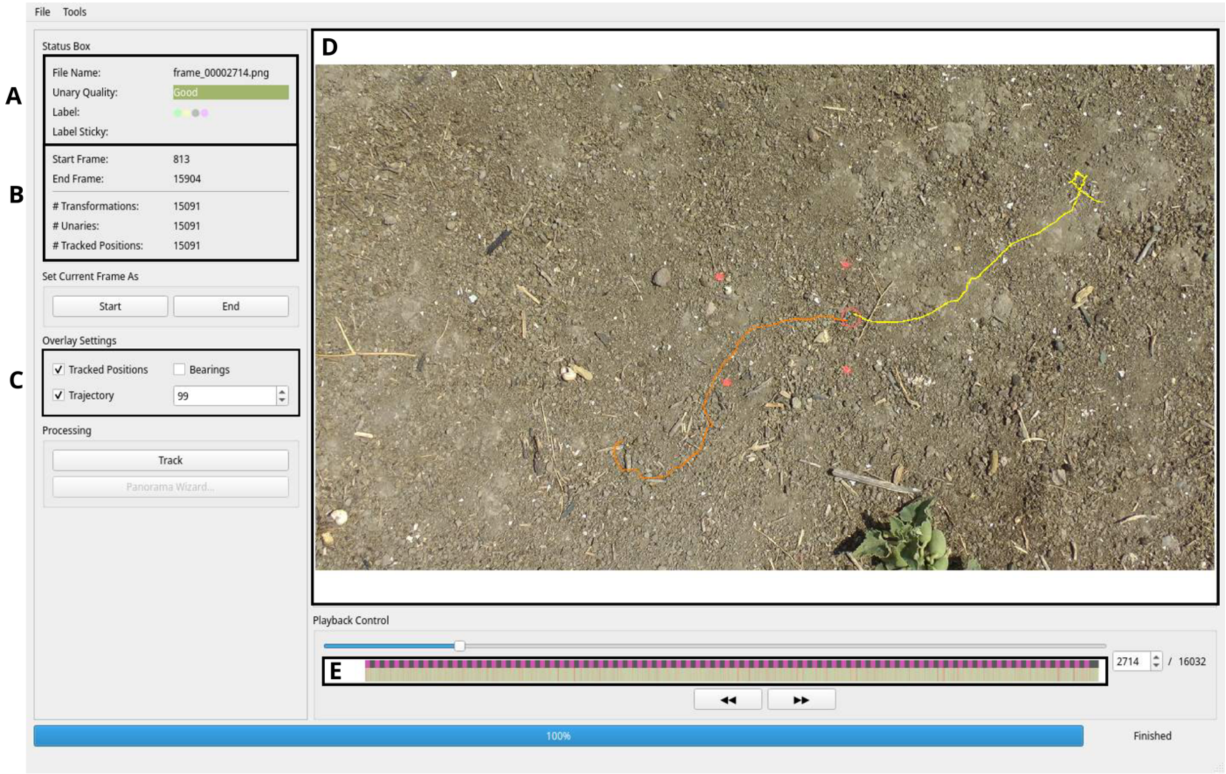

Fig. S2. ***CATER*** **software overview. (A)** Local info about current viewed image files, estimated quality for tracking and color-coded notation for current labels. **(B)** Global info about the full video, e.g., number of estimated transformations and tracked positions. **(C)** View settings controlling which items should be overlayed on the current view in **(D)**, e.g., current velocity vector (bearing). D: Image View showing current frame with requested overlay options. **(E)** Quality summary about each frame summarizing where manual corrections might be necessary. Alternating colors indicate chunks of frames that are optimized together when selecting parallel optimization.

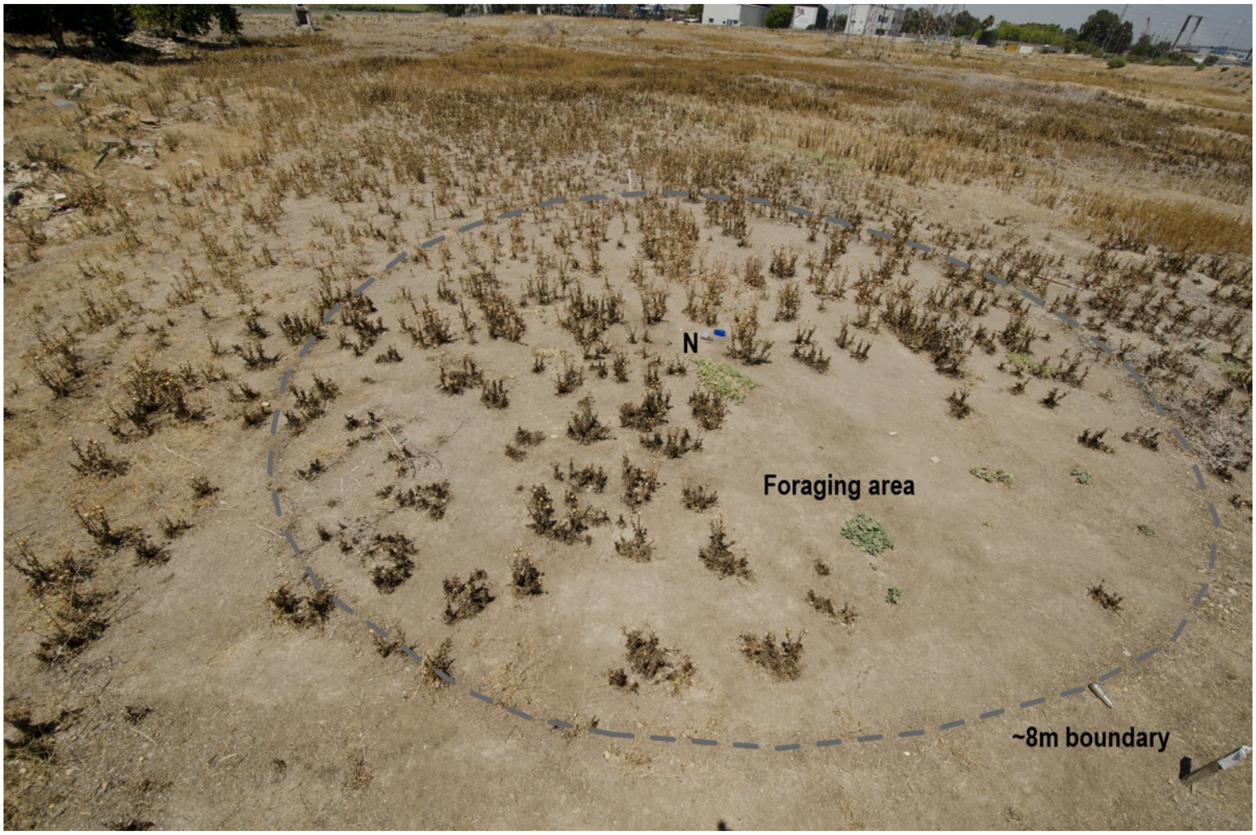

Fig. S3. Data capture site. All foragers belonged to the same colony of *Cataglyphis velox* ants located near Seville, Spain. Exit from the nest (N) was controlled using a plastic barrier (see blue ring), with the foraging area populated by vegetation providing a maze-like environment to navigate. The foraging area was swept of debris ahead of data capture to remove food and make tracking easier. Foragers were rewarded when they reached approximately 8m from the nest, which is visible by the edge of the swept area (dashed line).

Ant	Natural Foraging History										Displacement Tests					Notes
**1**	R1	R2	R3	R4	R5						ZVF	R6	ZVSF	R7	ZVOP	
**2**	R1	R2	R3	R4	R5	R6	R7	R8	R9	R10	ZVF	R11	ZVSF	R12	ZVOP	
**3**	R1	R2	R3	R4							ZVF	R5	ZVSF	R6	ZVOP	R1 to Feeder 1 (F1), R2 to F2, R3 to F3, , R5 to F3
**4**	R1	R2	R3	R4	R5	R6	R7				ZVF	R8	ZVSF	R9	ZVOP	
**5**	R1	R2	R3	R4	R5	R6	R7	R8	R9	R10	ZVF	R11	ZVSF	R12	ZVOP	R8 to F1, R9 & R10 to F2
**6**	R1	R2	R3	R4	R5						ZVF	R6	ZVSF	R7	ZVOP	R3 to F1, R4 & R5 to F2
**7**	R1	R2	R3	R4	R5	R6	R7	R8			ZVF	R9	ZVSF	R10	ZVOP	R7 to F1, R8 & R9 to F2 .
**8**	R1	R2	R3	R4							ZVF	R5	ZVSF			R2 to 8m+. R3 & R4 to F2 beyond 8m
**9**	R1	R2	R3	R4							ZVF	R5	ZVSF	R6	ZVOP	
**10**	R1	R2	R3	R4							ZVF	R5	ZVSF	R6	ZVOP	
**11**	R1	R2	R3	R4	R5	R6					ZVF	R7	ZVSF	R8	ZVOP	R3 to F1, R4 to F2, R5 & R6 to F3
**12**	R1	R2									ZVF	R3	ZVSF	R4	ZVOP	
**13**	R1	R2	R3	R4							ZVF	R5				
**14**	R1	R2	R3	R4	R5	R6	R7	R8			ZVF	R9	ZVSF			R6 to F1, R7 & R8 to F2

Key		
	x	Natural foraging path that did not reach 8m boundary
	x	Forging path in which ant found natural prey within the 8m area
	x	Foraging path that reached 8m boundary
	x	Completed displacement trials

Fig. S4. Summary table describing the video data included in the Ontogeny dataset. Data was collected for 14 ants from the same nest. R: Route; F: Feeder location; ZVF: Zero-Vector-Feeder (ant moved from nest back to feeder site); ZVSF: Zero-Vector-Semi-Familiar (ant moved from nest to location some meters orthogonal to its normal route); ZVOP: Zero-Vector-Opposite (ant moved from nest to a location in the opposite direction to the feeder site)

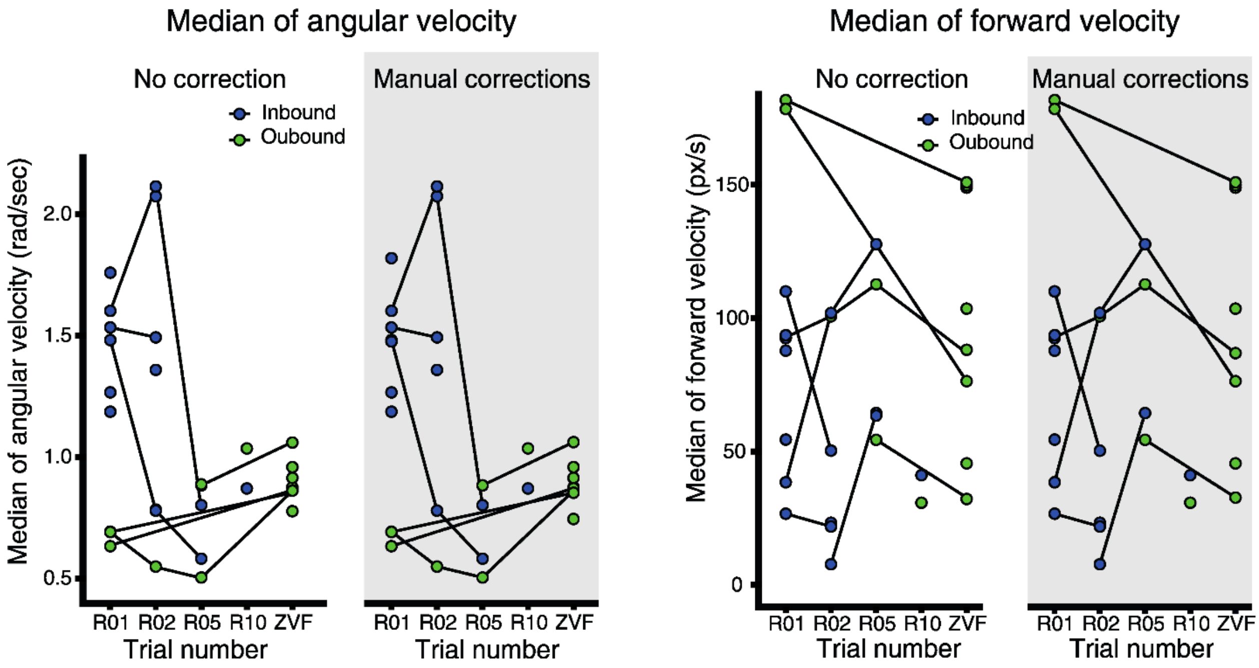

Fig. S5. Movement statistics. Analysis shows that statistics of movements such as the ants measured angular velocity (left) and forward velocity (right) do not change significantly if video tracking has been manually corrected (right, grey) or not (left, white). Each dot represents the trial of a given ants during an outbound trip (green) or homing trip (blue). Lines connects trials of same individuals. Trial ZVF indicates a ZV ant released at her familiar feeder.

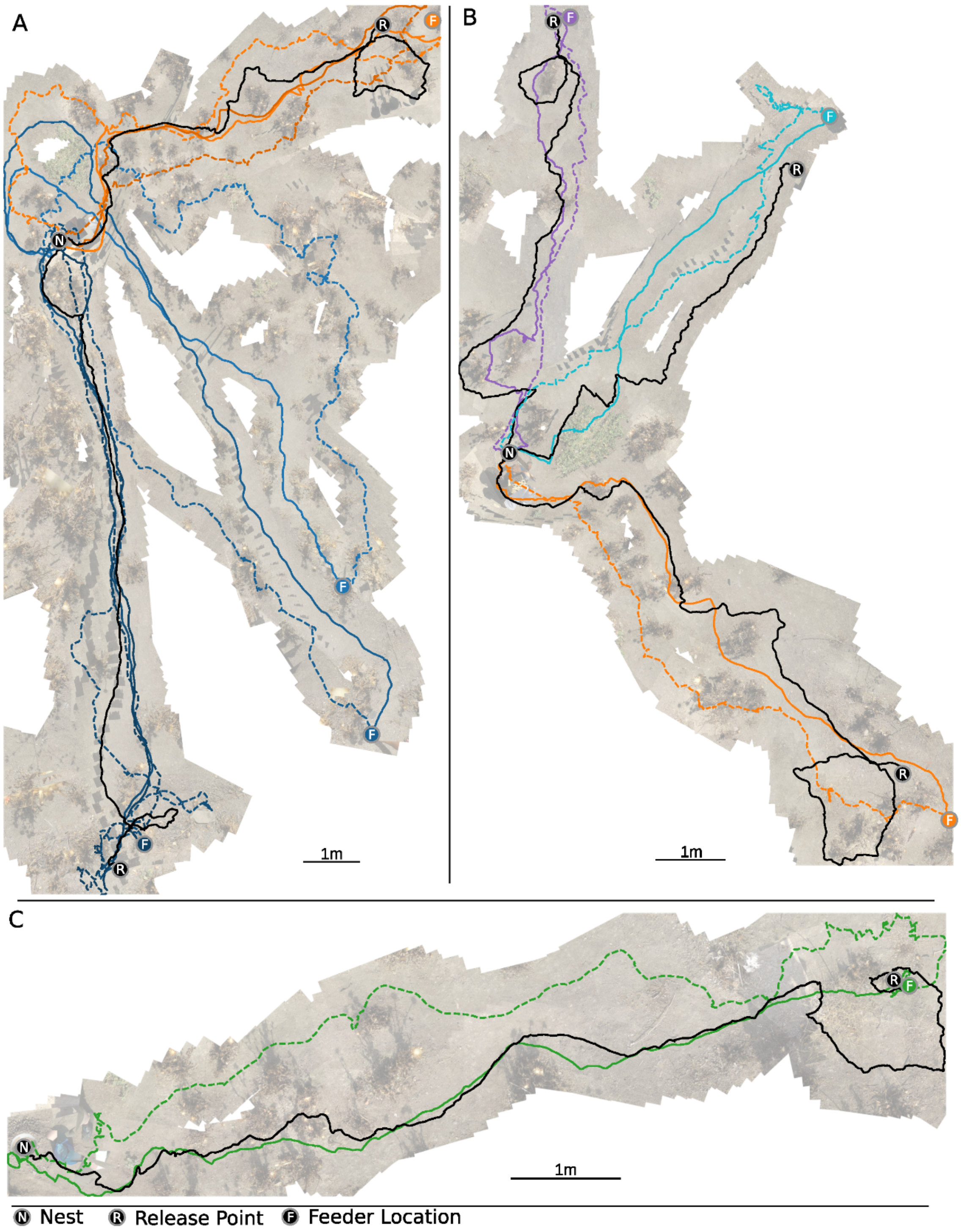

Fig. S6. Example ant routes and environment reconstructions. Three different examples where outward/inward paths are color coded according to the convention from the main text (each ant has its own color). Zero vector trails are always black, inward routes are solid, outward routes are dashed. **(A)** Two different ants where the blue ant has multiple different locations. **(B),(C)** Different ants with their last outward/inward path and the zero vector trial.

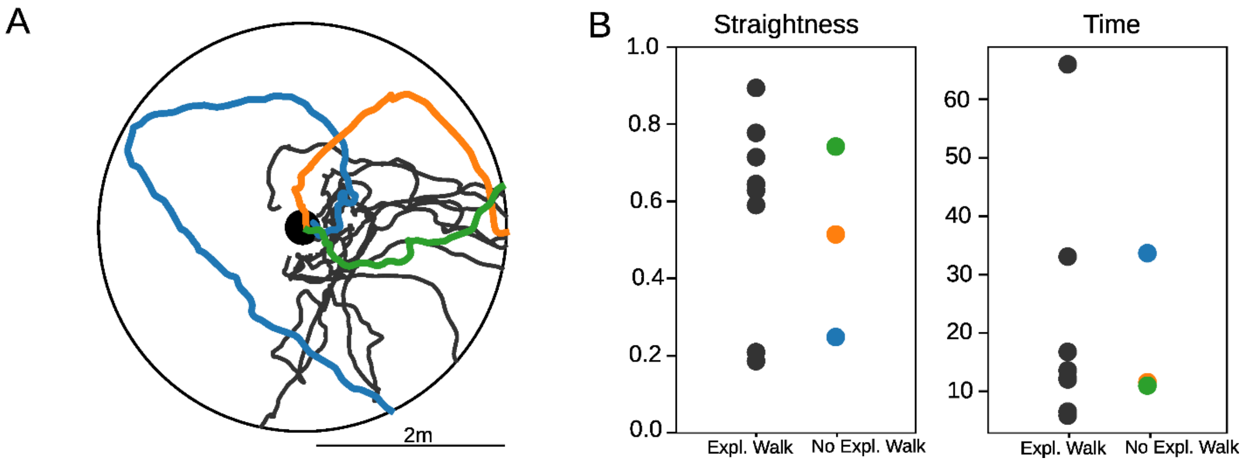

Fig. S7. Homing trajectories (A) and statistics (B). Assessment of homing capabilities of ants that did (black) and did not (color) perform exploratory walks before reaching 8m.

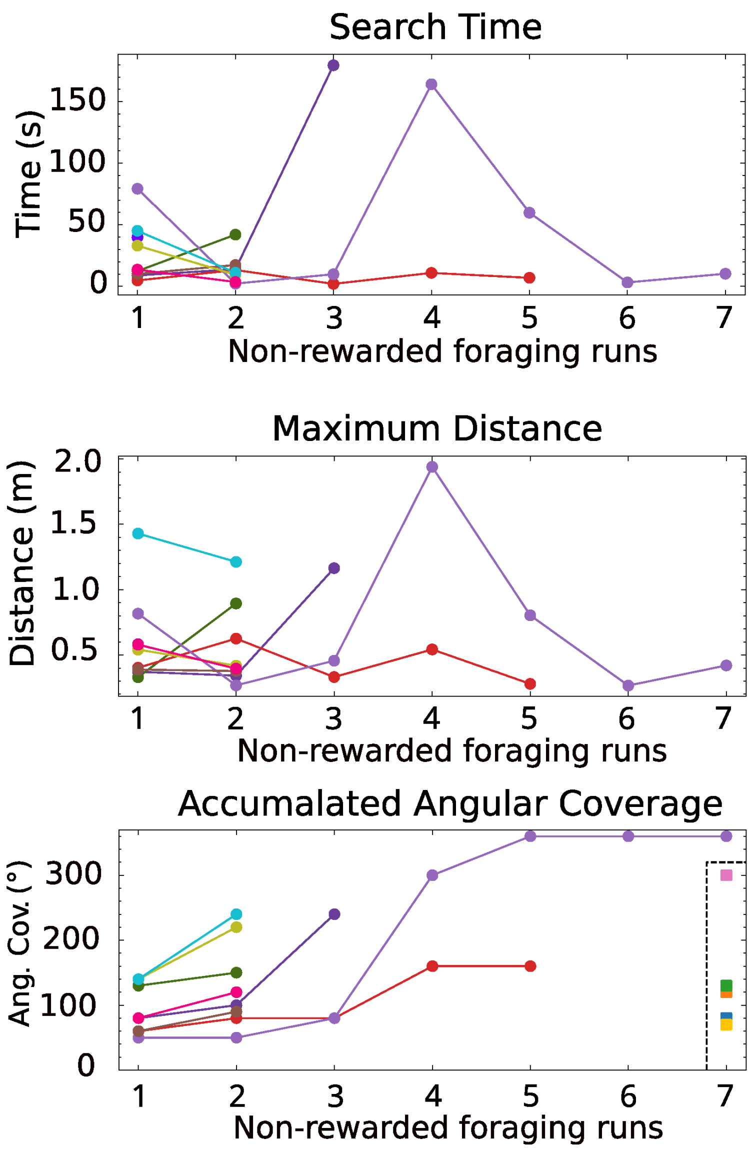

Fig. S8. Exploratory path statistics. Characterization of initial exploratory paths of foraging ants before reaching the 8m boundary.

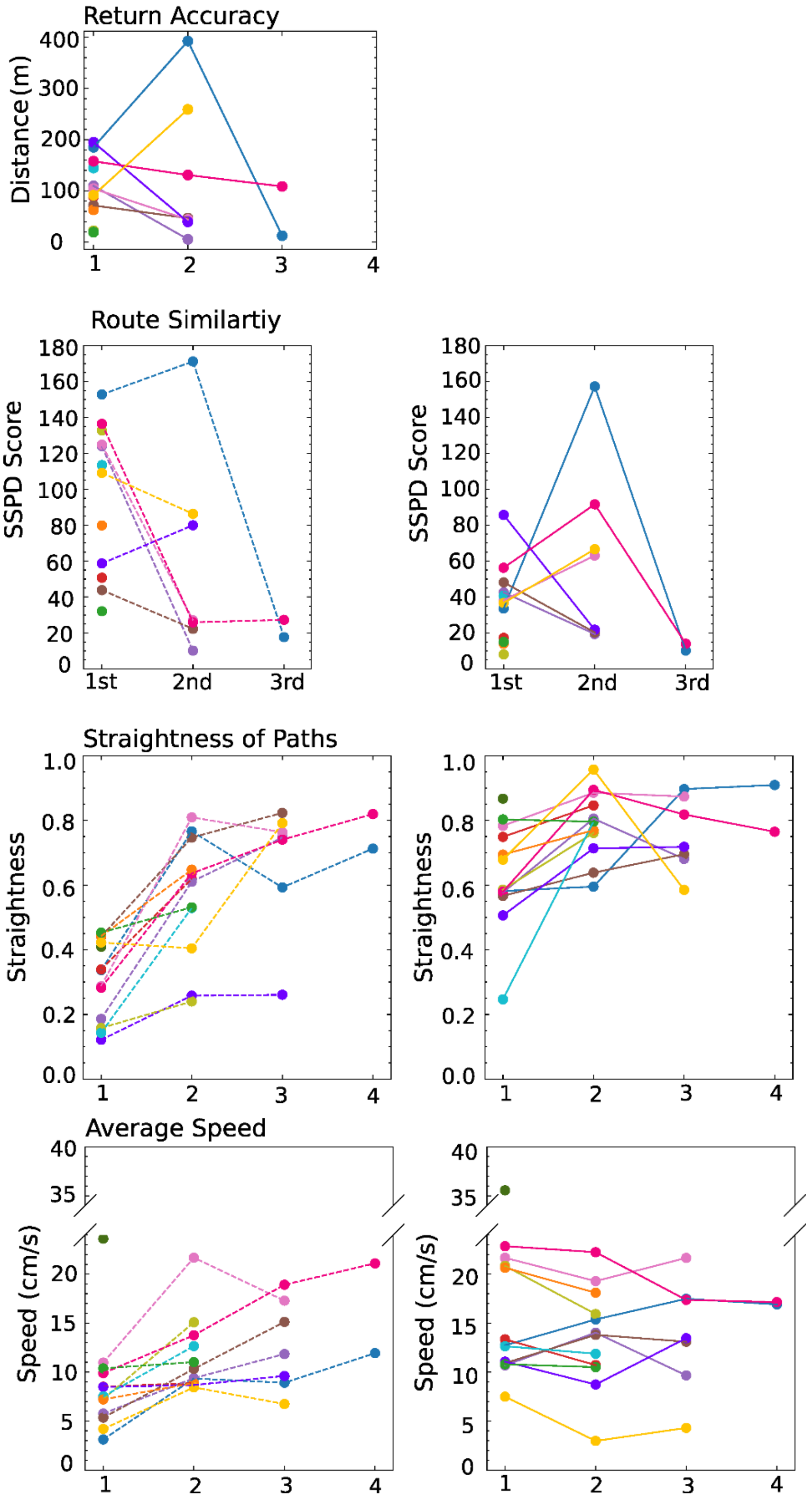

Fig. S9. Inward and outward paths statistics. Characteristics of the inward (left column, dashed lines) and outward paths (right column, solid lines) followed by ants (individual ants shown by a unique color across trials) once the 8m feeding site had been discovered.

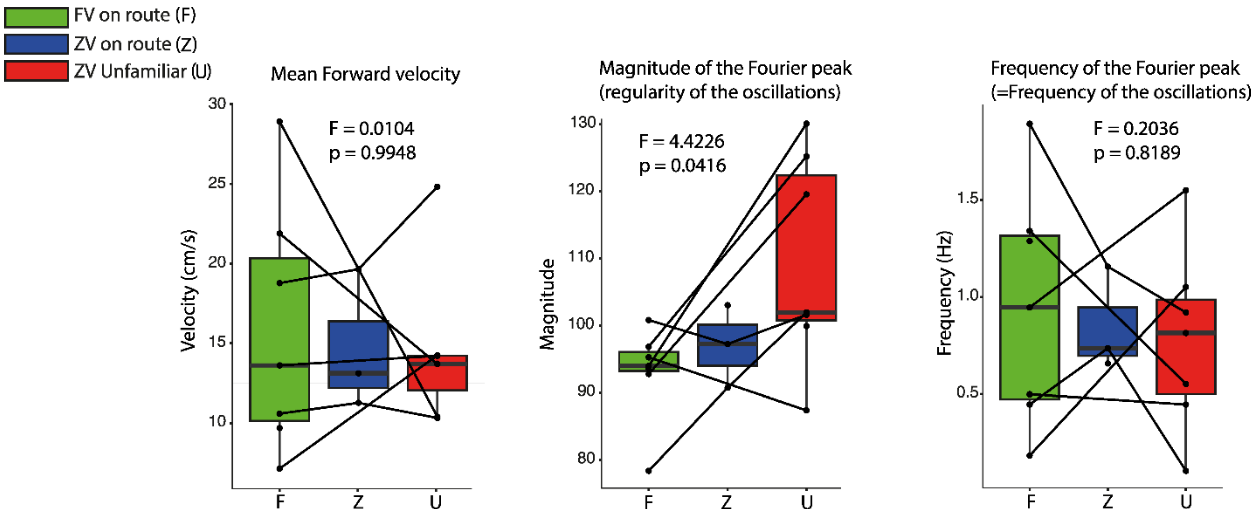

Fig. S10. Population level analysis. Population level analysis of motor parameters of experienced homing ants across three conditions: Full vector ants on their familiar route (green), zero-vector ants on their familiar route (blue) and ZV ants on unfamiliar terrain (red). Ants showed no significant differences in their mean forward velocities across conditions (left panel). Ants however displayed more regular oscillations in unfamiliar terrain (middle panel, significantly higher magnitude of Fourier's transform peak), but the frequency of these oscillations remained similar across conditions (right panel). Each dot represents the trial of a given ant; lines connect a same individual across conditions. Anova's F and p values are shown. Analysis was conducted only on homing ants that did not interact with bushes.

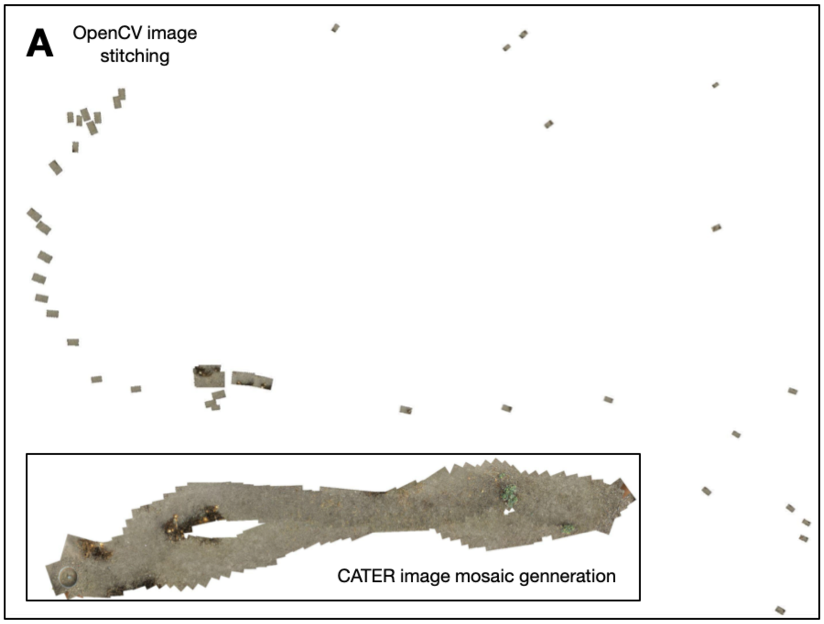

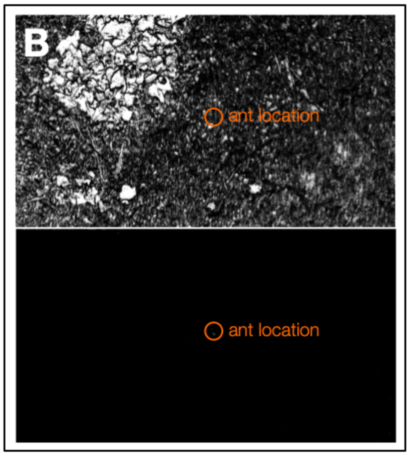

C

	CATER	YoloV5	Super-DiMP
med [pxl]	**12.2**	30.1	980.7
mad [pxl]	**8.1**	22.0	120.9

Fig. S11. Comparison of CATER to existing algorithms. (A) Comparison of the state-of-the-art OpenCV Image Stitcher in "Scan" Mode given the keyframes from our keyframe selection to our image stitching pipeline (inlay). Even with excessive parameter tuning the existing stitching algorithms fail to generate consistent image mosaics as environment reconstructions. **(B)** Comparison of different foreground extraction pipelines (ant location indicated). Top: kNN-based background subtraction as used in HyDAT (76). Note that the moving camera setting results in erroneous foreground estimates. Bottom: The camera-motion compensated foreground extraction as used in CATER results in more reliable sparse foreground estimates. **(C)** Comparison of CATER to two state-of-the-art object detection mechanisms, namely YOLOv5 (77) and Super-DiMP (78). Median error (med) and median absolute deviation (mad) are given in pixel.

Supplementary Tables

Table S1. Comparison of different detection and tracking systems as commonly used for animal tracking with reference to the three key challenges defined. Trackers marked by an asterisk (*) indicate trackers tested with our ant data; see Fig. S11 C.

	**Detecting small, occluded, low contrast animals**	**Camera-motion compensated trajectories**	**Environment reconstruction**
**CATER***	y *(med pixel error, 8.1)*	y *(dense global image registration)*	y *(2D image mosaic)*
**DeepLabCut (16)**	unknown *(feature-based, verified in lab data)*	n *(fixed camera only)*	n *(fixed camera only)*
**IDTracker.ai (21)**	unknown *(feature-based, verified in lab data)*	n *(fixed camera only)*	n *(fixed camera only)*
**YOLO v5* (78)**	y *(med pixel error, 22)*	n *(fixed camera only)*	n *(fixed camera only)*
**PATMOS (69)**	n (manual tracking)	y *(trajectories tracked using SLAM map)*	partial (Dense 3D map possible)
**SuperDiMP* (77)**	n *(med pixel error, 121)*	n *(fixed camera only)*	n *(fixed camera only)*
**HyDAT (76)**	y (built for stationary in-field settings)	n *(fixed camera only)*	n *(fixed camera only)*
**Ctrax (20)**	unknown *(feature-based, verified in lab data)*	n *(fixed camera only)*	n *(fixed camera only)*
**Francisco et al, 2020 (79)**	n (insufficient features for visual detection)	y (embedded in a SfM reconstruction)	y (projection into the SfM reconstruction)

Supplementary Movies

Movie S1. Video demonstrating tracking and image mosaic generation results. Top left: 3D visualization of the camera movement. Middle left: camera stream with ant detection (circle) trajectory (yellow line) overlay. Bottom left: speed profile of the ant. Middle image mosaic: Reconstruction result and ant trajectory. The black rectangle represents the current camera frustum and corresponds to the frames shown in the middle-left panel. The trajectory is color coded based on the velocity of the ant. Right: color coding legend (blue: low velocity, yellow: high velocity). The supplementary video can be found here:
https://cater.cvmls.org.

REFERENCES

1. South African Correspondent, Biotelemetry: Tracking of wildlife. *Nature* **234**, 508–509 (1971).

2. D. Tuia, B. Kellenberger, S. Beery, B. R. Costelloe, S. Zuffi, B. Risse, A. Mathis, M. W. Mathis, F. van Langevelde, T. Burghardt, R. Kays, H. Klinck, M. Wikelski, I. D. Couzin, G. van Horn, M. C. Crofoot, C. V. Stewart, T. Berger-Wolf, Perspectives in machine learning for wildlife conservation. *Nat. Commun.* **13**, 1 (2022).

3. S. E. R. Egnor, K. Branson, Computational analysis of behavior. *Annu. Rev. Neurosci.* **39**, 217–236 (2016).

4. N. C. Manoukis, T. C. Collier, Computer vision to enhance behavioral research on insects. *Ann. Entomol. Soc. Am.* **112**, 227–235 (2019).

5. B. G. Weinstein, A computer vision for animal ecology. *J. Anim. Ecol.* **87**, 533–545 (2018).

6. H. Dankert, L. Wang, E. D. Hoopfer, D. J. Anderson, P. Perona, Automated monitoring and analysis of social behavior in *Drosophila*. *Nat. Methods* **6**, 297–303 (2009).

7. A. D. Straw, K. Branson, T. R. Neumann, M. H. Dickinson, Multi-camera real-time three-dimensional tracking of multiple flying animals. *J. R. Soc. Interface* **8**, 395–409 (2011).

8. A. Gomez-Marin, N. Partoune, G. J. Stephens, M. Louis, automated tracking of animal posture and movement during exploration and sensory orientation behaviors. *PLOS ONE* **7**, e41642 (2012).

9. B. Risse, D. Berh, N. Otto, C. Klämbt, X. Jiang, FIMTrack: An open source tracking and locomotion analysis software for small animals. *PLOS Comput. Biol.* **13**, e1005530 (2017).

10. N. A. Swierczek, A. C. Giles, C. H. Rankin, R. A. Kerr, High-throughput behavioral analysis in *C. elegans*. *Nat. Methods* **8**, 592–598 (2011).

11. A. Pérez-Escudero, J. Vicente-Page, R. C. Hinz, S. Arganda, G. G. de Polavieja, idTracker: Tracking individuals in a group by automatic identification of unmarked animals. *Nat. Methods* **11**, 743–748 (2014).

12. S. L. Reeves, K. E. Fleming, L. Zhang, A. Scimemi, M-Track: A new software for automated detection of grooming trajectories in mice. *PLOS Comput. Biol.* **12**, e1005115 (2016).

13. A. Weissbrod, A. Shapiro, G. Vasserman, L. Edry, M. Dayan, A. Yitzhaky, L. Hertzberg, O. Feinerman, T. Kimchi, Automated long-term tracking and social behavioural phenotyping of animal colonies within a semi-natural environment. *Nat. Commun.* **4**, 2018 (2013).

14. V. Panadeiro, A. Rodriguez, J. Henry, D. Wlodkowic, M. Andersson, A review of 28 free animal-tracking software applications: Current features and limitations. *Lab Animal* **50**, 246–254 (2021).

15. J. M. Graving, D. Chae, H. Naik, L. Li, B. Koger, B. R. Costelloe, I. D. Couzin, DeepPoseKit, a software toolkit for fast and robust animal pose estimation using deep learning. *eLife* **8**, e47994 (2019).

16. A. Mathis, P. Mamidanna, K.M. Cury, T. Abe, V.N. Murthy, M.W. Mathis, M. Bethge, DeepLabCut: Markerless pose estimation of user-defined body parts with deep learning. *Nat. Neurosci.* **21**, 1281–1289 (2018).

17. T. D. Pereira, D. E. Aldarondo, L. Willmore, M. Kislin, S. S.-H. Wang, M. Murthy, J. W. Shaevitz, Fast animal pose estimation using deep neural networks. *Nat. Methods* **16**, 117–125 (2019).

18. J. Lauer, M. Zhou, S. Ye, W. Menegas, S. Schneider, T. Nath, M. M. Rahman, V. di Santo, D. Soberanes, G. Feng, V. N. Murthy, G. Lauder, C. Dulac, M. W. Mathis, A. Mathis, Multi-animal pose estimation, identification and tracking with DeepLabCut. *Nat. Methods* **19**, 496–504 (2022).

19. T. D. Pereira, N. Tabris, A. Matsliah, D. M. Turner, J. Li, S. Ravindranath, E. S. Papadoyannis, E. Normand, D. S. Deutsch, Z. Y. Wang, G. C. McKenzie-Smith, C. C.

Mitelut, M. D. Castro, J. D'Uva, M. Kislin, D. H. Sanes, S. D. Kocher, S. S.H. Wang, A. L. Falkner, J. W. Shaevitz, M. Murthy, SLEAP: A deep learning system for multi-animal pose tracking. *Nat. Methods* **19**, 486–495 (2022).

20. K. Branson, A. A. Robie, J. Bender, P. Perona, M. H. Dickinson, High-throughput ethomics in large groups of *Drosophila*. *Nat. Methods* **6**, 451–457 (2009).

21. F. Romero-Ferrero, M. G. Bergomi, R. C. Hinz, F. J. H. Heras, G. G. de Polavieja idtracker.ai: Tracking all individuals in small or large collectives of unmarked animals. *Nat. Methods* **16**, 179–182 (2019).

22. B. Q. Geuther, S. P. Deats, K. J. Fox, S. A. Murray, R. E. Braun, J. K. White, E. J. Chesler, C. M. Lutz, V. Kumar, Robust mouse tracking in complex environments using neural networks. *Commun. Biol.* **2**, 124 (2019).

23. Z. XU, X. E. Cheng, Zebrafish tracking using convolutional neural networks. *Sci. Rep.* **7**, 42815 (2017).

24. A. Gal, J. Saragosti, D. J. C. Kronauer anTraX, a software package for high-throughput video tracking of color-tagged insects. *eLife* **9**, e58145 (2020).

25. A. I. Dell, J. A. Bender, K. Branson, I. D. Couzin, G. G. de Polavieja, L. P.J.J. Noldus, A. Pérez-Escudero, P. Perona, A. D. Straw, M. Wikelski, U. Brose, Automated image-based tracking and its application in ecology. *Trends Ecol. Evol.* **29**, 417–428 (2014).

26. P. Karashchuk, J. C. Tuthill, B. W. Brunton, The DANNCE of the rats: A new toolkit for 3D tracking of animal behavior. *Nat. Methods* **18**, 460–462 (2021).

27. L. Haalck, M. Mangan, B. Webb, B. Risse, Towards image-based animal tracking in natural environments using a freely moving camera. *J. Neurosci. Methods* **330**, 108455 (2020).

28. M. Kristan, J. Matas, A. Leonardis, M. Felsberg, R. Pflugfelder, J.-K. Kämäräinen, H. J. Chang, M. Danelljan, L. Cehovin, Alan Lukežič, O. Drbohlav, J. Käpylä, G. Häger, S. Yan, J. Yang, Z. Zhang, G. Fernández, The ninth visual object tracking VOT2021 challenge

results, in *Proceedings of the IEEE/CVF International Conference on Computer Vision* (IEEE/CVF, 2021), pp. 2711–2738.

29. R. Wehner, in *Desert navigator: The Journey of an Ant* (Harvard University Press, Cambridge, 2020).

30. M. Kohler, R. Wehner, Idiosyncratic route-based memories in desert ants, melophorus bagoti: How do they interact with path-integration vectors? *Neurobiol. Learn. Mem.* **83**, 1–12 (2005).

31. M. Mangan, B. Webb, Spontaneous formation of multiple routes in individual desert ants (*Cataglyphis velox*). *Behav. Ecol.* **23**, 944–954 (2012).

32. A. Narendra, S. Gourmaud, J. Zeil, Mapping the navigational knowledge of individually foraging ants, *Myrmecia croslandi*. *Proc. Biol. Sci.* **280**, 20130683 (2013).

33. P. N. Fleischmann, R. Grob, R. Wehner, W. Rössler, Species-specific differences in the fine structure of learning walk elements in *Cataglyphis* ants. *J. Exp. Biol.* **220**, 2426–2435 (2017).

34. P. Jayatilaka, T. Murray, A. Narendra, J. Zeil, The choreography of learning walks in the Australian jack jumper ant *Myrmecia croslandi*. *J. Exp. Biol.* **221**, jeb185306 (2018).

35. T. Murray, Z. Kócsi, H. Dahmen, A. Narendra, F. L. Möel, A. Wystrach, J. Zeil, The role of attractive and repellent scene memories in ant homing (*Myrmecia croslandi*). *J. Exp. Biol.* **223**, jeb210021 (2020).

36. J. Zeil, P. N. Fleischmann, The learning walks of ants (*Hymenoptera:* Formicidae). *Myrmecological News* (2019).

37. A. Wystrach, F. Le Moël, L. Clement, S. Schwarz, A lateralised design for the interaction of visual memories and heading representations in navigating ants. *bioRxiv*, 2020.08.13.249193 (2020).

38. R. Hartley, A. Zisserman, in *Multiple view geometry in computer vision* (Cambridge University Press, Cambridge, 2011).

39. B. Risse, M. Mangan, L. D. Pero, B. Webb, Visual Tracking of Small Animals in Cluttered Natural Environments Using a Freely Moving Camera, in *Proceedings of the 2017 IEEE International Conference on Computer Vision Workshops* (ICCVW, 2017), pp. 2840–2849.

40. L. Haalck, B. Risse, Embedded Dense Camera Trajectories in Multi-Video Image Mosaics by Geodesic Interpolation-Based Reintegration, in *Proceedings of the IEEE/CVF Winter Conference on Applications of Computer Vision* (WACV, 2021), pp. 1849–1858.

41. R. Kays, M. C. Crofoot, W. Jetz, M. Wikelski, Terrestrial animal tracking as an eye on life and planet. *Science* **348**, aaa2478 (2015).

42. C. A. Freas, K. Cheng, Learning and time-dependent cue choice in the desert ant, *Melophorus bagoti*. *Ethology* **123**, 503–515 (2017).

43. C. A. Freas, M. L. Spetch, Terrestrial cue learning and retention during the outbound and inbound foraging trip in the desert ant, *Cataglyphis velox*. *J. Comp. Physiol. A Neuroethol. Sens. Neural Behav. Physiol.* **205**, 177–189 (2019),.

44. M. Müller, R. Wehner, Path integration provides a scaffold for landmark learning in desert ants. *Curr. Biol.* **20**, 1368–1371 (2010).

45. P. N. Fleischmann, M. Christian, V. L. Müller, W. Rössler, R. Wehner, Ontogeny of learning walks and the acquisition of landmark information in desert ants, *Cataglyphis fortis*. *J. Exp. Biol.* **219**, 3137–3145 (2016).

46. T. S. Collett, J. Zeil, Insect learning flights and walks. *Current Biology* **28**, R984–R988 (2018).

47. B. Baddeley, P. Graham, P. Husbands, A. Philippides, A model of ant route navigation driven by scene familiarity. *PLoS Comput. Biol.* **8**, e1002336 (2012).

48. A. Wystrach, M. Mangan, A. Philippides, P. Graham, Snapshots in ants? New interpretations of paradigmatic experiments. *J. Exp. Biol.* **216**, 1766–1770 (2013).

49. A. D. Dewar, A. Philippides, P. Graham, What is the relationship between visual environment and the form of ant learning-walks? An in silico investigation of insect navigation. *Adapt. Behav.* **22**, 163–179 (2014).

50. W. Stürzl, J. Zeil, N. Boeddeker, J. M. Hemmi, How wasps acquire and use views for homing. *Curr. Biol.* **26**, 470–482 (2016).

51. F. Le Moël, A. Wystrach, Opponent processes in visual memories: A model of attraction and repulsion in navigating insects' mushroom bodies. *PLoS Comput. Biol.* **16**, e1007631 (2020).

52. S. E. Pfeffer, S. Bolek, H. Wolf, M. Wittlinger, Nest and food search behaviour in desert ants, *Cataglyphis*: A critical comparison. *Anim. Cogn.* **18**, 885–894 (2015).

53. P. Ardin, F. Peng, M. Mangan, K. Lagogiannis, B. Webb, Using an insect mushroom body circuit to encode route memory in complex natural environments. *PLOS Comput. Biol.* **12**, e1004683 (2016).

54. X. Sun, S. Yue, M. Mangan, A decentralised neural model explaining optimal integration of navigational strategies in insects. *eLife* **9**, e54026 (2020).

55. A. Wystrach, A. Philippides, A. Aurejac, K. Cheng, P. Graham, Visual scanning behaviours and their role in the navigation of the Australian desert ant *Melophorus bagoti*. *J. Comp. Physiol. A* **200**, 615–626 (2014).

56. D. D. Lent, P. Graham, T. S. Collett, Phase-dependent visual control of the zigzag paths of navigating wood ants. *Curr. Biol.* **23**, 2393–2399 (2013).

57. L. Clement, S. Schwarz, A. Wystrach, An intrinsic oscillator underlies visual navigation in ants. *bioRxiv*, 2022.04.22.489150 (2022).

58. J. R. Riley, U. Greggers, A. D. Smith, D. R. Reynolds, R. Menzel, The flight paths of honeybees recruited by the waggle dance. *Nature* **435**, 205–207 (2005).

59. A. Kodzhabashev, M. Mangan, Route Following Without Scanning, in *Biomimetic and Biohybrid Systems*, S. Wilson, P. Verschure, A. Mura, T. Prescott, Eds. (Springer, Cham, 2015), vol. 9222

60. S. Toledo, D. Shohami, I. Schiffner, E. Lourie, Y. Orchan, Y. Bartan, R. Nathan, Cognitive map–based navigation in wild bats revealed by a new high-throughput tracking system. *Science* **369**, 188–193 (2020).

61. T. Hafting, M. Fyhn, S. Molden, M.-B. Moser, E. I. Moser, Microstructure of a spatial map in the entorhinal cortex. *Nature* **436**, 801–806 (2005).

62. T. D. Pereira, J. W. Shaevitz, M. Murthy, Quantifying behavior to understand the brain. *Nat. Neurosci.* **23**, 1537–1549 (2020).

63. M. Reiser, The ethomics era? *Nat. Methods* **6**, 413–414 (2009).

64. M. B. Sokolowski, *Drosophila*: Genetics meets behaviour. *Nat. Rev. Gen.* **2**, 879–890 (2001).

65. R. Hajar, Animal testing and medicine. *Heart Views* **12**, 42 (2011).

66. R. Schiffman, Drones flying high as new tool for field biologists. *Science* **344**, 459 (2014).

67. B. Kellenberger, M. Volpi, D. Tuia, Fast Animal Detection in UAV Images Using Convolutional Neural Networks, in *Proceedings of the 2017 IEEE International Geoscience and Remote Sensing Symposium* (IGARSS, 2017), pp. 866–869.

68. V. Raoult, L. Tosetto, J. E. Williamson, Drone-based high-resolution tracking of aquatic vertebrates. *Drones* **2**, 37 (2018).

69. P. C. Brady, Three-dimensional measurements of animal paths using handheld unconstrained GoPro cameras and VSLAM software. *Bioinspir. Biomim.* **16**, 026022 (2021).

70. B. Millward, S. Maddock, M. Mangan, CompoundRay, an open-source tool for high-speed and high-fidelity rendering of compound eyes. *eLife* **11**, e73893 (2022)

71. R. Goulard, C. Buehlmann, J. E. Niven, P. Graham, B. Webb, A unified mechanism for innate and learned visual landmark guidance in the insect central complex. *PLOS Comput. Biol.* **17**, e1009383 (2021).

72. L. Schad, J. Fischer, Opportunities and risks in the use of drones for studying animal behaviour. *Methods Ecol. Evol.*, 10.1111/2041-210X.13922 (2022).

73. A. Savitzky, M. J. E. Golay, Smoothing and differentiation of data by simplified least squares procedures. *Anal. Chem.* **36**, 1627–1639 (1964).

74. E. Batschelet, in *Circular Statistics in Biology* (Academic Press, Cambridge, 1981).

75. P. Besse, B. Guillouet, J.-M. Loubes, F. Royer, Review and perspective for distance-based clustering of vehicle trajectories. *IEEE Trans. Intell. Transp. Syst.* **17**, 3306–3317 (2016).

76. M. Ratnayake, A. Dyer, A. Dorin, Tracking individual honeybees among wildflower clusters with computer vision-facilitated pollinator monitoring. *PLOS ONE* **16**, e0239504 (2021).

77. G. Bhat, M. Danelljan, L. Van Gool, R. Timofte, Learning Discriminative Model Prediction for Tracking, in *Proceedings of the 2019 IEEE/CVF International Conference on Computer Vision* (ICCV, 2019)

78. G. Jocher, YOLOv5 by Ultralytics, https://github.com/ultralytics/yolov5 (2020)

79. F. Francesco, P. Nührenberg, A. Jordan, High-resolution, non-invasive animal tracking and reconstruction of local environment in aquatic ecosystems. *Mov. Ecol.* **8**, 27 (2020)
